# Study of microstructure and corrosion behavior of nano-Al_2_O_3_ coating layers on TiO_2_ substrate via polymeric method and microwave combustion

**DOI:** 10.1038/s41598-024-68566-6

**Published:** 2024-08-08

**Authors:** H. K. Abd El-Hamid, A. A. Gaber, Rehab E. A. Ngida, H. E. H. Sadek, R. M. Khattab, Howida S. Mandour

**Affiliations:** 1 Refractories, Ceramics and Building Materials Department, National Research Centre (NRC), El-Buhouth St., Dokki, 12622 Cairo Egypt; 2 Physical Chemistry Department, National Research Centre (NRC), El-Buhouth St., Dokki, 12622 Cairo Egypt; 3 Pharos University, Canal El Mahmoudia Street, Smouha, Alexandria, Egypt

**Keywords:** Ceramics, Titania, Nano-alumina, Polymeric, Microwave, Corrosion, Bioinorganic chemistry, Biochemistry, Chemistry, Materials science

## Abstract

The study describes the successful development of a TiO_2_ ceramic substrate with a protective nano-Al_2_O_3_ coating using two different coating techniques: microwave combustion and polymeric methods. The coated ceramics demonstrate enhanced corrosion resistance compared to the uncoated substrate. The optimal TiO_2_ substrate was prepared by firing it at 1000 °C. This was done to give the desired physical properties of the TiO_2_ substrate for the coating procedures. Nano-Al_2_O_3_ powder was coated onto the surface of the TiO_2_ substrates. The TiO_2_ substrates with the Al_2_O_3_ coating were then calcined (heat-treated) at 800 and 1000 °C. The structures, morphology, phase composition, apparent porosity, bulk density, and compressive strength of the substrate and coated substrate were characterized. Upon firing at 1000 °C, it was discovered that the two phases of TiO_2_—rutile and anatase—combine in the substrate. Once the substrate has been coated with nano Al_2_O_3_ at 1000 °C, the anatase is transferred into rutile. When compared to the substrate, the coated substrate resulted in a decrease in porosity and an increase in strength. The efficiency of the ceramic metal nanoparticles Al_2_O_3_ as a good coating material to protect the TiO_2_ substrates against the effect of the corrosive medium 0.5 M solution of H_2_SO_4_ was measured by two methods: potentio-dynamic polarization (PDP) and the electrochemical impedance spectroscopy (EIS). The results indicated that the corrosion rate was decreased after the substrate coated with alumina from (67.71 to 16.30 C.R. mm/year) and the percentage of the inhibition efficiency recorded a high value reaching (78.56%). The surface morphology and composition after electrochemical measurements are investigated using SEM and EDX analysis. After conducting the corrosion tests and all the characterization, the results indicated that the coated TiO_2_ substrate prepared by the polymeric method at 800 °C displayed the best physical, mechanical, and corrosion-resistant behavior.

## Introduction

Corrosion damage can occur to many metal structures, including bridges, automobiles, aeroplanes, industrial buildings, desalination plants, pharmaceuticals, and more, which lowers their efficiency and shortens their useful or productive life^1^. One of these causes of the metallic structure becoming weaker, resulting in material failures and structural degradation, is corrosion. Because ceramic materials corrode far less quickly than metals, they might be regarded as corrosion-resistant materials in comparison^2,3^. Distinct classes of ceramic materials (silicate, oxide, and non-oxide ceramic) exhibit distinct corrosion and corrosion resistance types. Although metal corrosion is an electrochemical process, the degree of ceramic corrosion is determined by the material's solubility. Low corrosion rates are primarily caused by the chemical composition and the microstructure^4,5^.

Different ceramic coating materials, such as Al_2_O_3_, ZrO_2_, WC-12% Co, and TiO_2_, prevent corrosion^4–6^. Zirconia-ceria-benzotriazole ceramic-based hybrid coating's morphological evaluation by S. M. Dezfuli and M. Sabzi reveals a very homogenous and uniform coating devoid of cracks after an immersion time of 48 h. This is because the zirconia-ceria-benzotriazole ceramic-based coating has a self-healing mechanism, which has improved corrosion resistance^7^. Aluminum oxide, Al_2_O_3_, more commonly known as alumina, is a very significant ceramic material with numerous technological applications^4^. Its unique properties include high hardness, chemical inertness, wear resistance, and a high melting point. Alumina ceramics are widely used in many refractory materials, high-temperature bearings, various mechanical parts, and critical components in chemical process environments where materials are subjected to aggressive chemical attack, increasingly higher temperatures, and pressures. This is due to the excellent properties of alumina ceramics. Microstructural findings by M. Sabzi and S. M. Dezfuli revealed that the deposition of Al_2_O_3_ ceramic film on copper-based heterostructured resulted in the formation of α-Al_2_O_3_ and CuAl_2_O_4_ phases and enhanced corrosion resistance in Cu/Al_2_O_3_ copper-based coatings at all immersion times^8^. The corrosion resistance of alumina ceramics in aqueous acidic solution (HCl, H_2_SO_4_, H_3_PO_4_) under hydrothermal conditions was reported by Schachat et al.^9^. The researchers discovered that the sequence of the acids' decreasing corrosive effects on alumina ceramics is H_3_PO_4_ > HCl > H_2_SO_4_.

A titanium oxide that occurs naturally is called titanium dioxide (TiO_2_). It is also known as titania or titanium (IV) oxide. Having a molecular mass of 79.86 g/mol, a density of 3.9–4.2 g/cm^3^, a refractive index in the range of 2.5–2.75, and a Mohs hardness of 5.5–7, TiO_2_ is an inexpensive and widely accessible white oxide ceramic^10,11^. Rutile, anatase, and brookite are the three crystalline forms it can take. While brookite has an orthorhombic structure, rutile and anatase have tetragonal structures. Only the anatase and rutile phases of TiO_2_ are utilized in industrial applications^10,11^. TiO_2_ is non-toxic, biocompatible, flammable-free, chemically and photochemically stable, and a semiconductor^10–12^. Researchers M. Sabzia and S.H. Mousavi Anijdan have discovered a uniform heterostructured NiO-TiO_2_ film on an AA7050 aluminum alloy that is appropriate and homogeneous for use in solar absorption plates^13^. To give anti-wear and corrosion-resistant characteristics, TiO_2_ is frequently deposited as thin films or thick film coatings^10,12^. This shielding layer is known as TiO_2_ and is also quite stable. However, titanium's poor tribological characteristics pose a serious application challenge, particularly when strong wear resistance is desired. In addition to halide anions, the titanium's generated oxide coatings are also susceptible to deterioration by sulphate solutions and other media^14^. Another investigation discovered that when coupled with TiO_2_, Al_2_O_3_ improves mechanical properties such as hardness and fracture toughness^15–17^.

Nanomaterials have just been introduced as a successful method to lessen corrosion. Nanomaterials are substances with at least one morphological characteristic on the nanoscale (less than 100 nm), such as grain size, particle size, structure size, etc.^9^. Nanoparticles, nanotubes, nanowires, and nano-rods are examples of one-dimensional objects, whereas nano-platelets, nano-sheets, and nano-films are examples of two-dimensional objects. Improved thermal, mechanical, physical, chemical, magnetic, electronic, and optical properties can be found in nanomaterials^18^. Their small diameters enable higher volume fractions at the surfaces and, hence, higher interaction areas^18^. Corrosion can be efficiently hindered by nanocoating materials because of the uniform physical barrier they consistently establish on a material's surface. ZrO_2_, TiO_2_, Al_2_O_3_, SiO_2_, and their composites are the best anti-corrosion materials for this use^1^.

Physical vapor deposition (PVD), chemical vapor deposition (CVD), electrochemical deposition, thermal spraying, plasma spraying, spin-, dip-, spray-coating, and sol–gel methods are the most widely used techniques for creating the nano-protective coated layer^19–21^. Additionally, the polymeric (Pechini) and microwave combustion methods are widely used to synthesize the nano-powder coatings on the substrate surface. The Pechini method has many advantages, such as its simplicity and low-temperature requirements, which prevent potential decomposition problems; its capacity to produce high-purity, high-quality, and stoichiometric coatings; and its simplicity in adjusting the film thickness^21,22^. Additionally, microwave combustion is the most recent preparation method because it requires a simple, quick, and low-energy approach for synthesizing nano-metal oxide materials. You can say nano-oxide particles or nano-materials^23,24^.

This work concerns the synthesis and characterization of nano-Al_2_O_3_ coating layers on the TiO_2_ substrate surface; the phase composition, physical, microstructure, and corrosion resistance for the substrate and the deposited coated layers are well studied and discussed. The coated layers are prepared in nano-form by utilizing two different synthesis techniques, the microwave combustion method and the polymeric method, to obtain a novel body with stable and uniformly coated bodies with good corrosive resistant behavior to extend the ceramic applications.

## Materials and methods

### Starting materials

The starting materials used in this work are TiO_2_ (Alpha Chemika) and AlCl_3_. 6H_2_O (Arabian Medical & Scientific Lab. Sup, Co.), Ethylene glycol, and citric acid were supplied by Merk-Schuchardt and Aldrich, respectively. Urea was supplied by Alpha Chemika.

### TiO_2_ substrate samples preparation

TiO_2_ substrate samples were created and compressed under a pressure of 18 MPa to create the TiO_2_ substrate samples. The samples are sintered for a full hour at different temperatures of 900, 1000, and 1100 °C.

### Preparation of TiO_2_ substrates coated by alumina prepared by polymeric method (Pechini method)

The polymeric (Pechini) method is based on the polymerization of metallic citrate with ethylene glycol. An aqueous solution containing hydrocarboxylic acid, such as citric acid, was utilized to chelate cations. An organic ester is created when a polyalcohol, like ethylene glycol, is added. Heating-induced polymerization creates a homogenous resin with evenly dispersed metal ions throughout the organic matrix.

In a beaker with 20 ml of water, a specific amount of AlCl_3_. 6H_2_O is dissolved. Citric acid and ethylene glycol are added to this water volume in a 60:40 mass percent ratio.

The calculated amount of citric acid was dissolved in 10ml of millipore water followed by constant stirring at 60 to 70 °C. A clear and homogenous sol solution of citric acid was prepared. Once added, the salt, AlCl_3_.6H_2_O (12.11 g), is continuously stirred until completely dissolved. Finally, the calculated dose of ethylene glycol was added with continuous stirring^22^. The first part of the freshly prepared solution is used for the coating procedure. The other part is subjected to drying at 100 °C, followed by firing for an hour at different temperatures, 800 °C and 1000 °C, to obtain the nano-alumina powder to undergo the powder investigations.

The prepared coating solution is deposed upon the titania substrate surface by dipping the TiO_2_ substrates in the freshly prepared coating solution for two hours; the coating solution containing the substrates is suctioned. The coated substrate samples are then taken out of the sol solution, dried in the air at 100 °C, and then fired for an hour at two different temperatures: 800 °C and 1000 °C. The coated substrates' deposition, drying, and calcination procedures took place twice to obtain a uniform covered alumina coating layer on the titania substrate's surface.

### Preparation TiO_2_ substrates coated by alumina prepared via microwave combustion method

The microwave combustion method includes preceding salt (AlCl_3_. 6H_2_O) added to the urea fuel to create the combustion solution. Millipore water was employed as a solvent during the preparation of samples. To create a uniform solution, the compounds were first dispersed individually in millipore water and blended for about an hour at room temperature. The inorganic components in the precursors functioned as the oxidizers in the combustion process, while urea served as the fuel. There was a 2:1 M ratio between the fuel (urea) and the oxidizer (AlCl_3_. 6H_2_O) (F/O). A particular amount of AlCl_3_. 6H_2_O is dissolved in 20 ml of water in a beaker and then mixed with the calculated weight of urea^23^. The first part of the freshly prepared solution is used for the coating procedure. In contrast, the other part is subjected to calcination at 800 °C and 1000 °C after exposure to dried air at 100 °C to obtain the nano-Al_2_O_3_ powder prepared via microwave for powder investigations.

**Figure 1:**
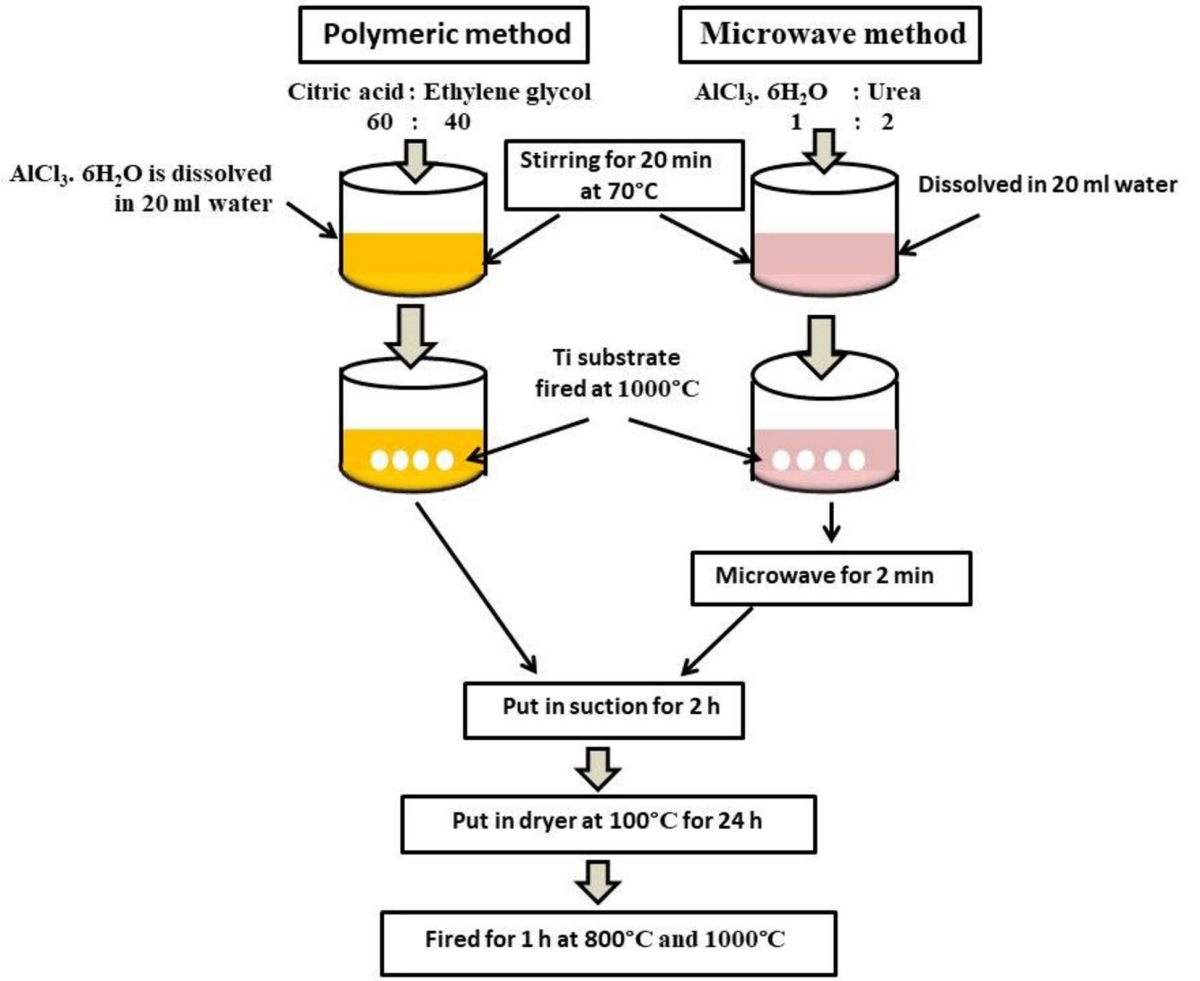
Steps of coating process by polymeric method and microwave combustion.

The deposition procedure of the prepared alumina coating solution on the titania substrates surfaces took place by dipping the TiO_2_ substrates in the prepared alumina homogenous solutions (pre-solution) in silica crucible and placed inside a microwave oven and exposed to 1000 W of electricity at 300 °C for two minutes at a frequency of 2.45 GHz. The coated TiO_2_ substrates in solution are removed from the microwave and put under suction for two hours. The microwave synthesized coated titania substrates by alumina layer are then removed from the solution and dried in the air for 1 h, first at 100 °C and then fired at two different temperatures: 800 °C and 1000 °C. The coated substrates' dipping, microwave irradiation step, drying, and calcination procedures were taken twice to obtain uniform covered alumina coating layers on the titania substrate's surface. The two coating methods are illustrated in Table 1 and graphically represented in Fig. 1.

**Table 1:** The sequence of coating process parameters.

Steps no.	Polymeric method	Microwave combustion
1	Added citric acid: ethylene glycol with a percentage of 60:40, respectively	Added urea : AlCl_3_.6H_2_O with percentage 2:1 respectively
2	Amount of AlCl_3_. 6H_2_O is dissolved in distilled water and added with continuous stirring	Amount of AlCl_3_. 6H_2_O is dissolved in distilled water and then mixed with the calculated weight of urea
3	Continuous stirring of the sol. at 60 to 70 °C until it reaches a clear sol	
6	The freshly prepared sol. used for dipping the Ti substrate is put in the microwave for 2 min, as in the microwave method	
7	The coating sol. Containing Ti substrates are suctioned for 2 h	
8	After that, the coated substrate was removed from the sol. And dried at 100 °C for 24h	
9	Then, it fired for an hour at 800 and 1000 °C	
10	The deposition, drying, and calcination procedures took place twice to obtain a uniform alumina covering layer

### Characterization

X-ray diffraction (XRD) was carried out to examine phase composition using monochromatic Cu K radiation (D 500, Siemens, Mannheim, Germany). Moreover, Fourier transform infrared spectra were examined by a Japanese Jasco-300E FTIR spectrophotometer. The synthesized samples were crushed, and the fine powders were combined in a 1:100 ratio with KBr. The particle size was examined using a transmission electron microscope (TEM) model JEOL JEM-2100 outfitted with an x-ray energy-dispersive spectroscopy system and a high-angle angular dark-field detector. The structure and morphology of the materials were ascertained using Scanning Electron Microscopy (SEM) observations and FEI, QUANTA FEG, 250. According to ASTM C373-88, apparent porosity and bulk density were calculated using the Archimedes principle^25^. A LLOYD device, Model LR 10K, was utilized to study the compressive strength of samples using the specific method in^26^

The detection of the corrosion process took place in 0.5M solution of H_2_SO_4_ for the prepared discs of uncoated TiO_2_ substrates and for coated TiO_2_ substrates by nano- Al_2_O_3_ layers, which were prepared by two different techniques: microwave and polymeric methods at two different temperatures; 800 and 1000 °C. The detection of the corrosion process was carried out by using a potentiostat/galvanostat with electrochemical impedance spectroscopy Autolab PGSTAT302N. The electrochemical cell was configured with three electrodes: Ag/AgCl electrode as the reference electrode, platinum electrode as the counter electrode, and the coated and uncoated TiO_2_ substrates acting as the working electrode. Open circuit potential (OCP), potentiodynamic polarization (PDP), and electrochemical impedance spectroscopy (EIS) are the electrochemical methods that were used to measure corrosion behavior. The potentials of the corrosion process are recorded concerning the reference electrode (Ag/AgCl).

## Results and discussion

### Characterization of TiO_2_ substrate samples

#### Physical properties of TiO_2_ substrate samples

**Figure 2:**
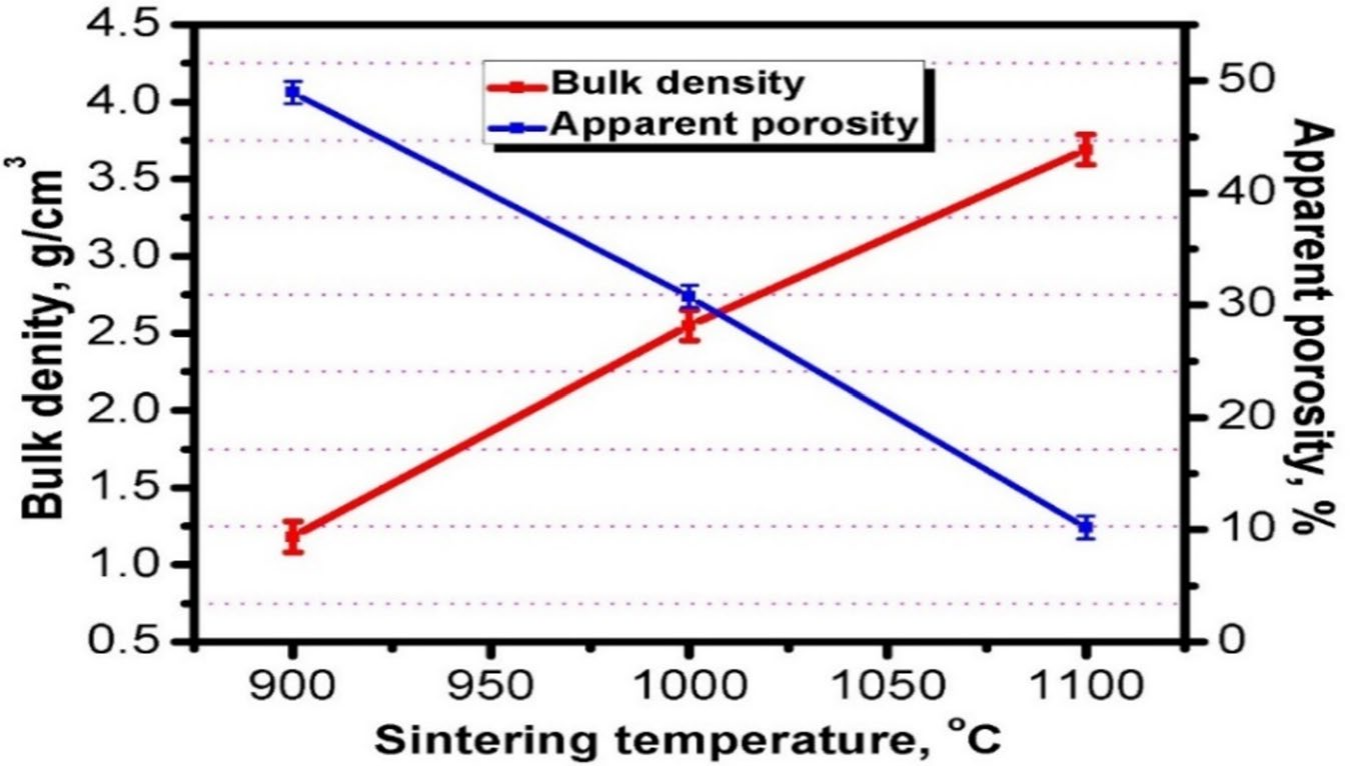
The effect of sintering temperatures on the bulk density and apparent porosity of TiO_2_ substrate.

The physical properties are indicated in terms of bulk density and apparent porosity. The effect of different sintering temperatures at 900, 1000, and 1100 °C on the apparent porosity and bulk density of the TiO_2_ substrate sample are clearly shown in Fig. 2. As demonstrated in Fig. 2, the apparent porosity reduced, and bulk density increased by increasing the sintering temperature. The apparent porosities at 900, 1000, and 1100 °C are 48.98, 30.76, and 10.20%, respectively. The bulk densities are 1.18, 2.55 and 3.69 g/cm^3^ at 900, 1000 and 1100 °C. In this study, it is important to prepare titania substrate structure in a porous form to allow penetration of nano-coating solution through the pores of the substrate to facilitate adhesion to its body followed by complete covering of its surface by nano-alumina layers to increase the corrosive resistance of the coated titania substrates to a high extent^27^. Accordingly, the TiO_2_ substrate samples fired at 1000 °C were selected as the optimal substrates to be applied for the coating procedures.

#### Phase composition of TiO_2_ substrate sample

**Figure 3:**
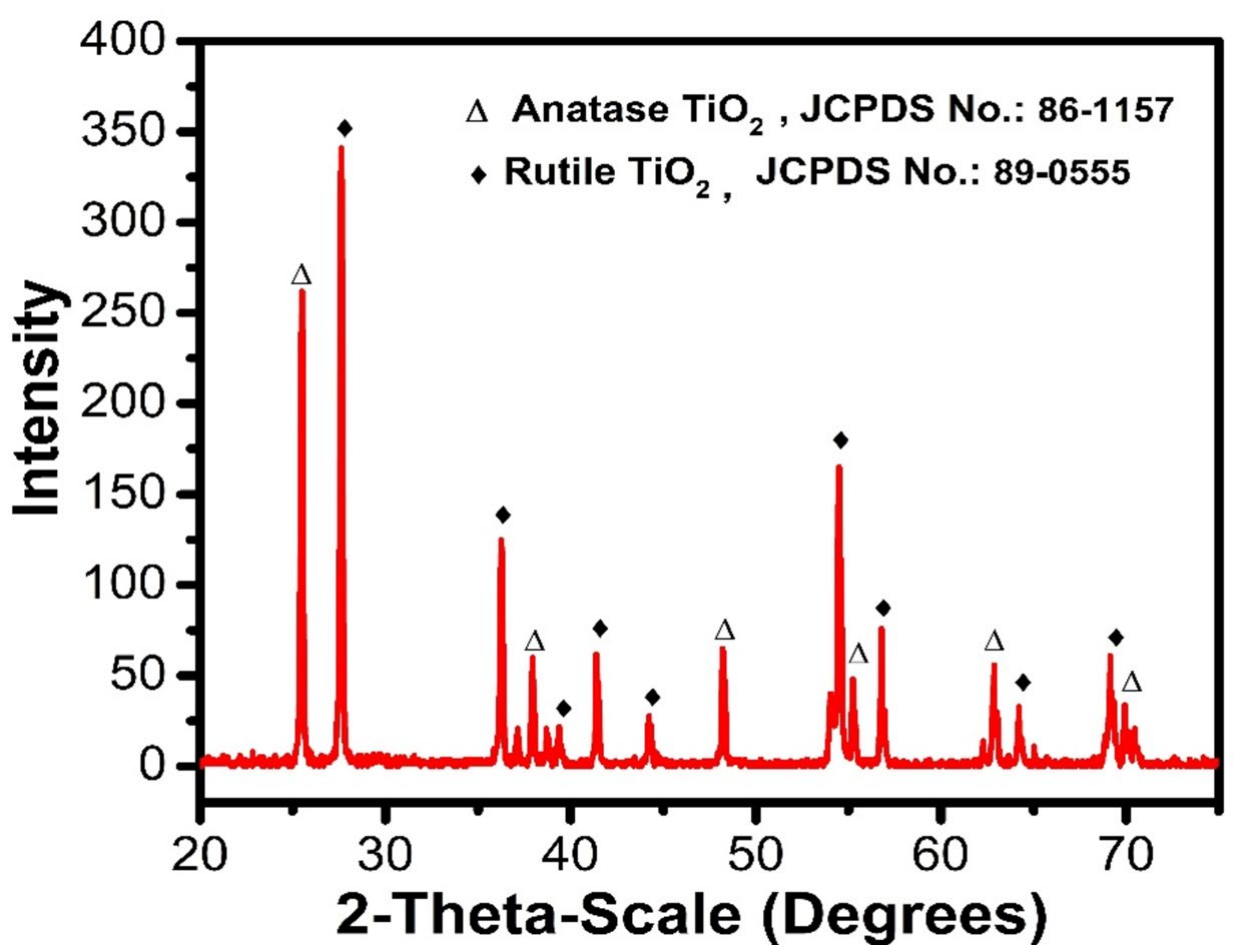
XRD pattern of TiO_2_ substrate sintered at 1000 °C.

Figure 3 depicts the sintered TiO_2_ substrate sample's powder XRD pattern. The anatase and rutile polymorph-related peaks' locations were extracted from PDF #86–1157 and #89–0555, respectively. Anatase and rutile, which are both TiO_2_ phases, are combined. The primary peak is at 25.3°, which corresponds to the crystal plane (101) for the TiO_2_ anatase phase and the crystal plane (110) for the TiO_2_ rutile phase at 27.4°^28^. Generally, the synthesis process used in preparing the titania powder precursors significantly impacts the phase transition from anatase to rutile, varying from 850 °C in the sol–gel approach to 1100 °C in the solid-state reaction^29^.

#### IR analysis of TiO_2_ substrate sample

**Figure 4:**
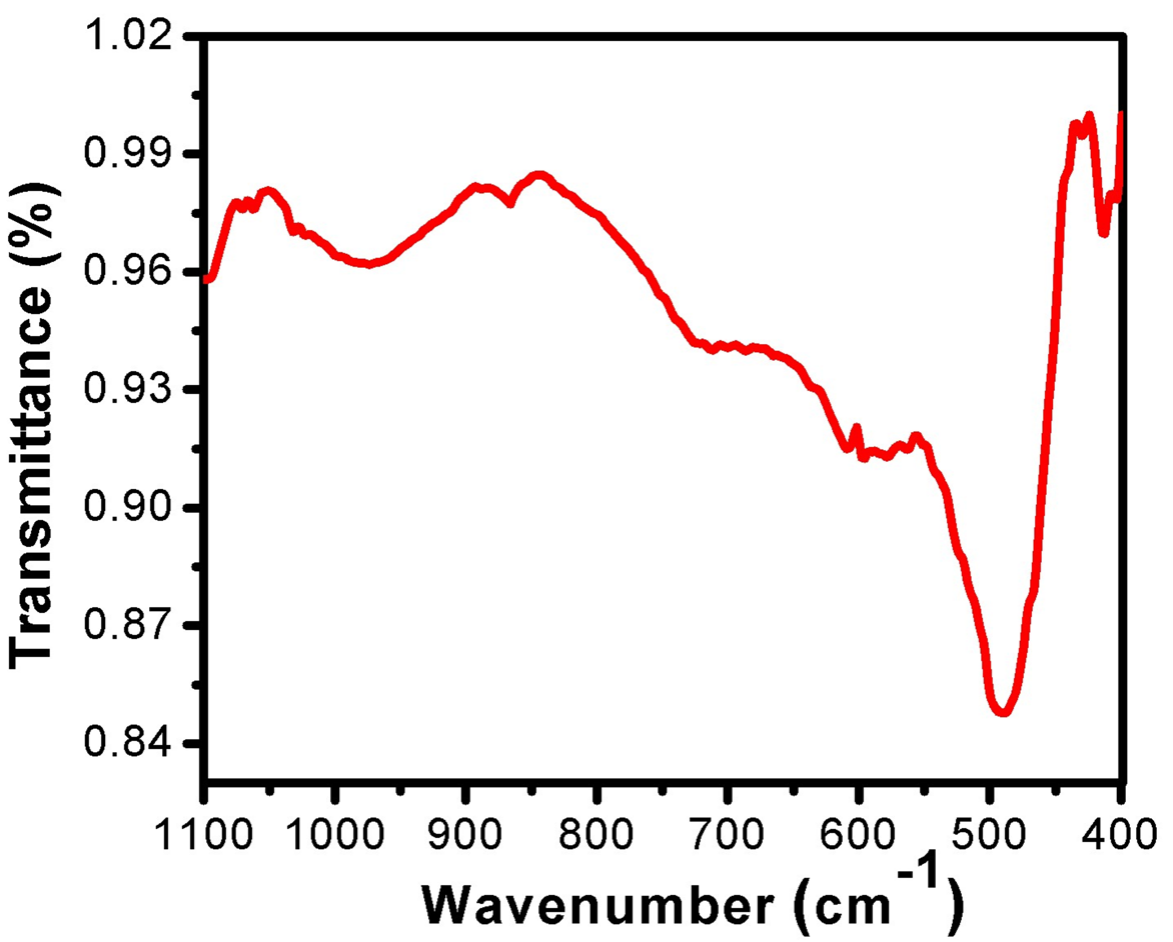
FTIR spectra spectrum of TiO_2_ substrate sintered at 1000 °C.

Figure 4 shows the FTIR spectra of the substrate TiO_2_ samples sintered at 1000 °C according to XRD results. Signals between 400 and 1000 cm^−1^ correspond to Ti–O–Ti vibration^30^. Abroad absorption band between 450 and 800 cm^−1^ regions is ascribed to the vibration assigned to absorption of the Ti–O-Ti linkages in TiO_2_ particles^30^. For the pure TiO_2_, the peak at 420 cm^−1^ is related to the presence of anatase titania. According to the standard spectra of TiO_2_, the peak at 499 and 850 cm^−1^ may be or was attributed to the vibration of the Ti–O bond in the TiO_2_ (rutile titania) lattice. The small IR absorption band at 870 cm^−1^ is attributed to the Ti–O-Ti stretching vibrations^31^.

#### SEM of TiO_2_ substrate sample

**Figure 5:**
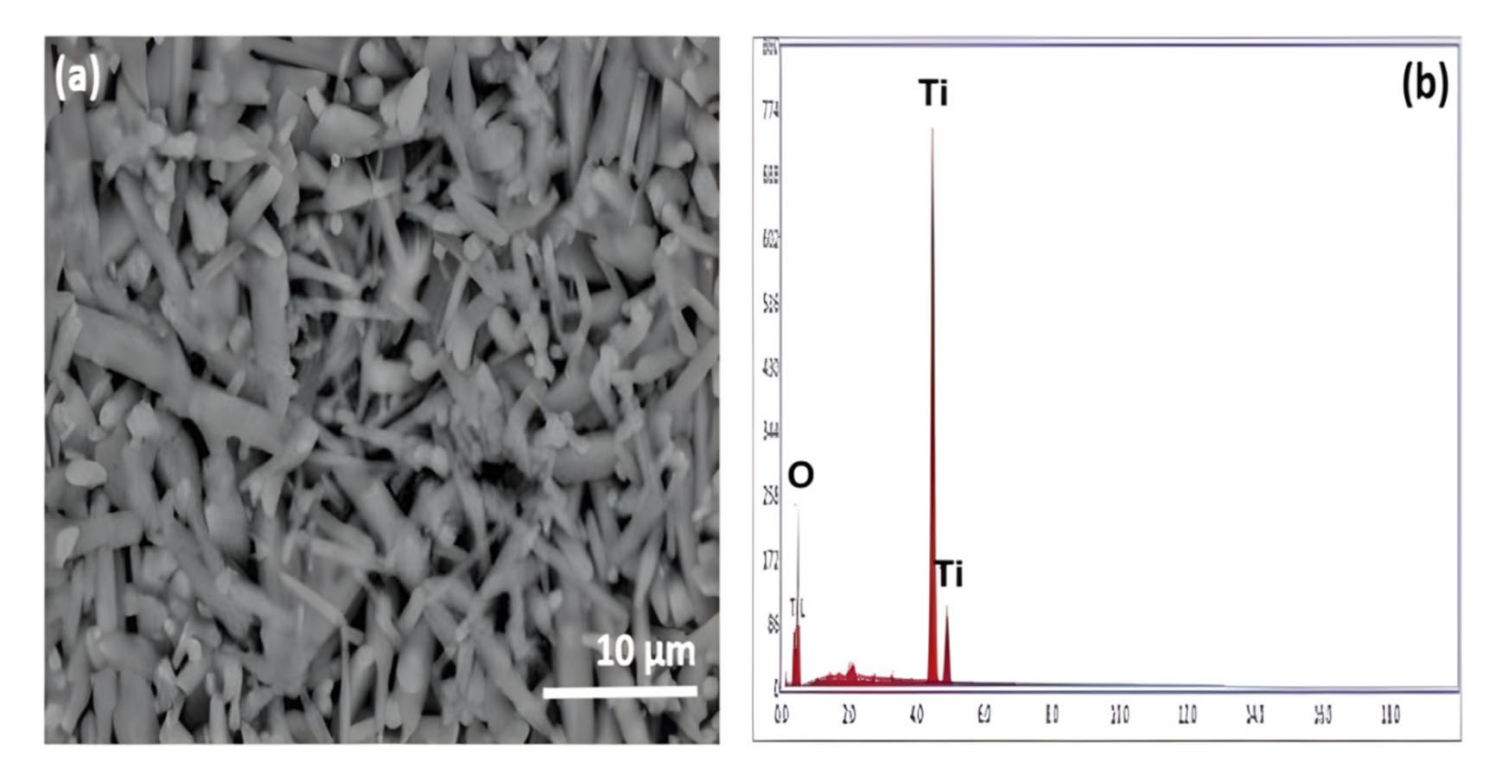
SEM image and corresponding EDS spectra of TiO_2_ substrate sintered at 1000 °C.

The morphology of the TiO_2_ substrate is seen in Fig. 5a after calcination of the sample at 1000 °C. The SEM images demonstrate the TiO_2_ particles appeared in spherical and rod shapes. Rod-like anatase particles were covered by small spherical particles of rutile^32^. The particle sizes of rod TiO_2_ shapes ranged between 4 to 15 µm. The spherical particles of TiO_2_ ranged from 100nm to 2µm. EDS spectra spectrum shown in Fig. 5b showed that only Ti and O elements were detectable, and no other elements were identified for the samples studied. The uniformity of the synthesized TiO_2_ consists of well-interconnected crystallite. The percentage of the surface porosity of the prepared titania substrate was calculated to be about (30%). Thus, some pinholes can be observed in the sample surface.

#### TEM analysis of TiO_2_ substrate sample

**Figure 6:**
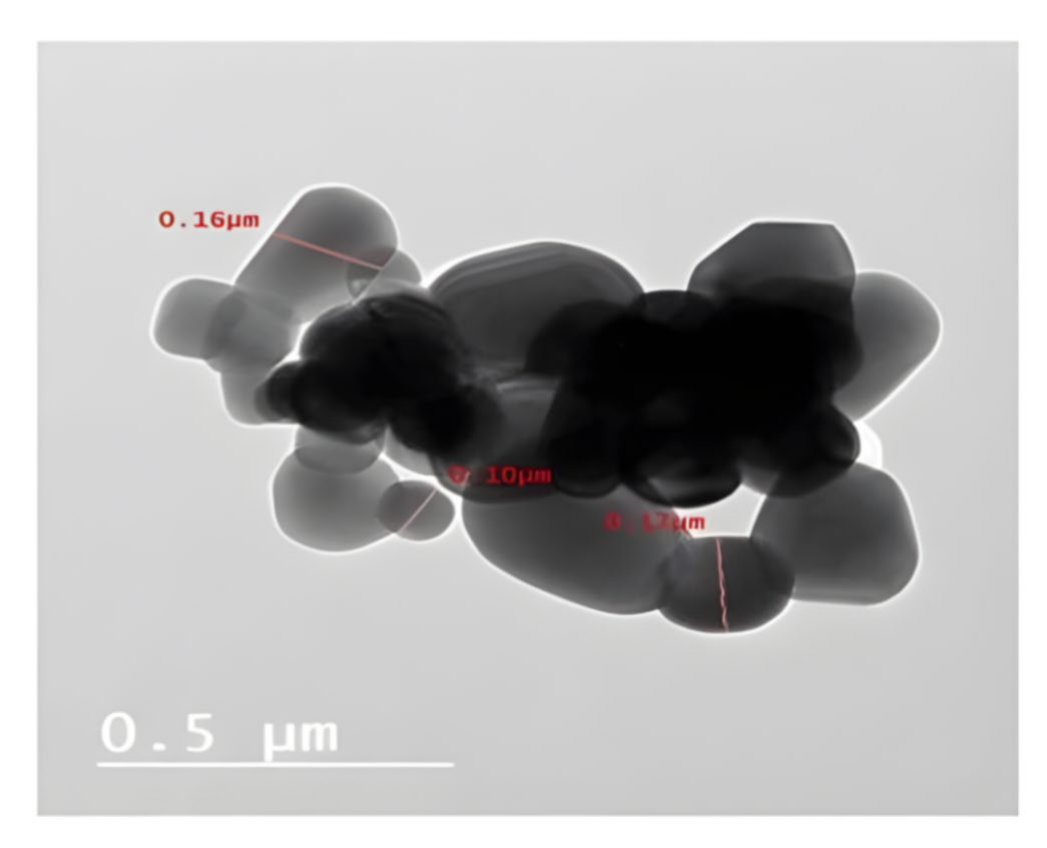
TEM photomicrograph of TiO_2_ substrate sintered at 1000 °C.

TEM of powder TiO_2_ material fired at 1000 °C is shown in Fig. 6. It is indicated by the well crystallization of TiO_2_ particles with octahedral geometry; as shown in Fig. 6, the geometry of the rutile form of titanium dioxide is twisted hexagonal with a size ranging between 0.10 to 0.17µm.

### Characterization of Al_2_O_3_ powder prepared via polymeric and microwave techniques

#### Phase composition of Al_2_O_3_ powder

**Figure 7:**
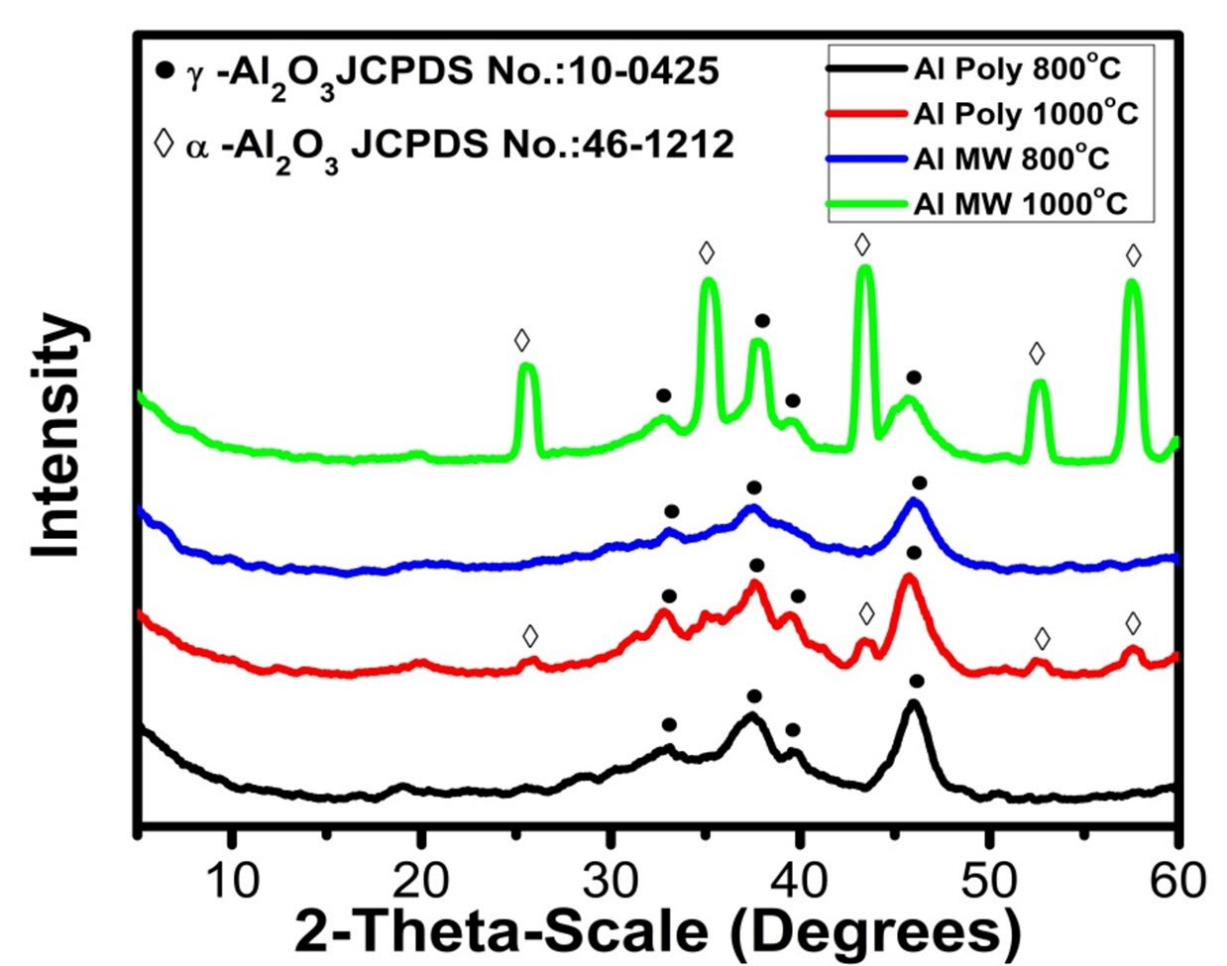
XRD patterns of Al_2_O_3_ powder prepared by polymeric (Poly) and microwave methods (MW) at 800 °C and 1000 °C.

XRD of the fired alumina powders prepared by the polymeric method and microwave combustion method at different temperatures is shown in Fig. 7. It was observed that the alumina powders calcined at 800 °C that were prepared by both methods are presented in their gamma phase (γ-Al_2_O_3_) as indicated by card No. PDF #10-0425. Upon increasing the temperature to 1000 °C, the small amount of corundum phase (α-Al_2_O_3_) is indicated according to card No. PDF# 46-1212 beside the main gamma alumina phase (γ-Al_2_O_3_) for the alumina powder prepared by polymeric method (Poly). On the contrary, alumina's main corundum phase (α-Al_2_O_3_) is obtained using the microwave combustion method (MW). Thus, the microwave method is more favored in increasing the crystallinity degree of the prepared alumina, which is indicated by a decrease in the γ-Al_2_O_3_ phase peak intensity^23^.

#### IR analysis of Al_2_O_3_ powder

**Figure 8:**
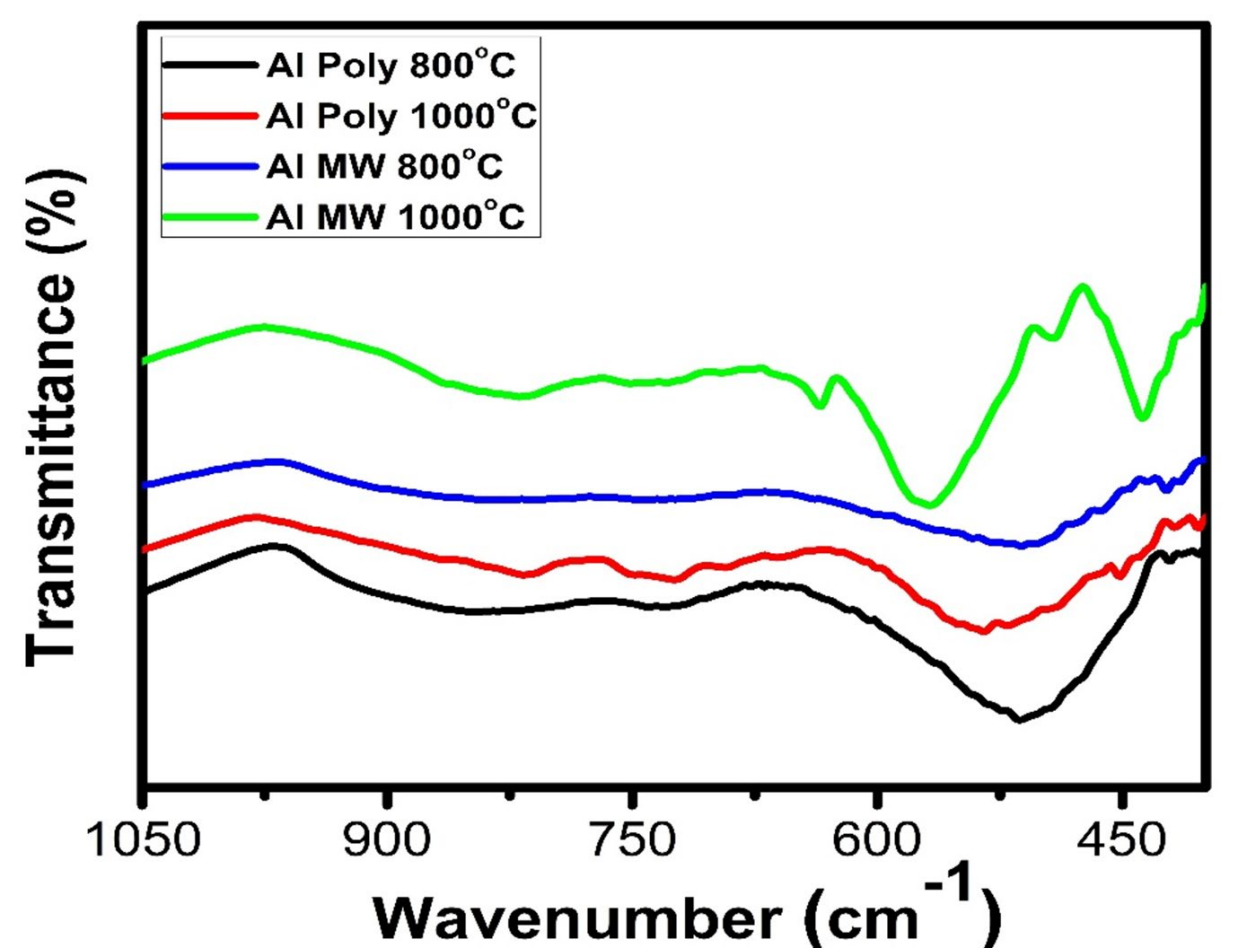
FTIR spectra of Al_2_O_3_ powder prepared by polymeric (Poly) and microwave methods (MW) at 800 °C and 1000 °C.

The FTIR spectra of prepared alumina powders calcined at 800 and 1000 °C and prepared by polymeric method (Poly) and microwave method (MW), respectively, are shown in Fig. 8. It was observed that alumina powders calcined at 800 °C showed an appearance of γ-Al_2_O_3_. The calcined alumina powder prepared by polymeric method showed sharp bands corresponding to the stretching vibration of Al-O appearing at 720 and 513 cm^–1^ while they appeared at 812, 740, and 436 cm^–1^ for the alumina calcined powder prepared by microwave method^16^. Bands for α-Al_2_O_3_ appear at 639, 576, and 437 cm^–1^ for powders calcined at 1000 °C and prepared by microwave method. Meanwhile, bands around 1084 and 1095 cm^–1^ are assigned to the symmetric bending of Al–O for calcined powders at 800 and 1000 °C, respectively^16^. From these results, it was observed that the band intensity of the alpha alumina at 1000 °C is high and appeared at 437 cm^–1^ compared with the calcined samples at 800 °C that were prepared by both polymeric and microwave methods; these results are confirmed by the XRD results.

#### TEM analysis of Al_2_O_3_ powder

**Figure 9:**
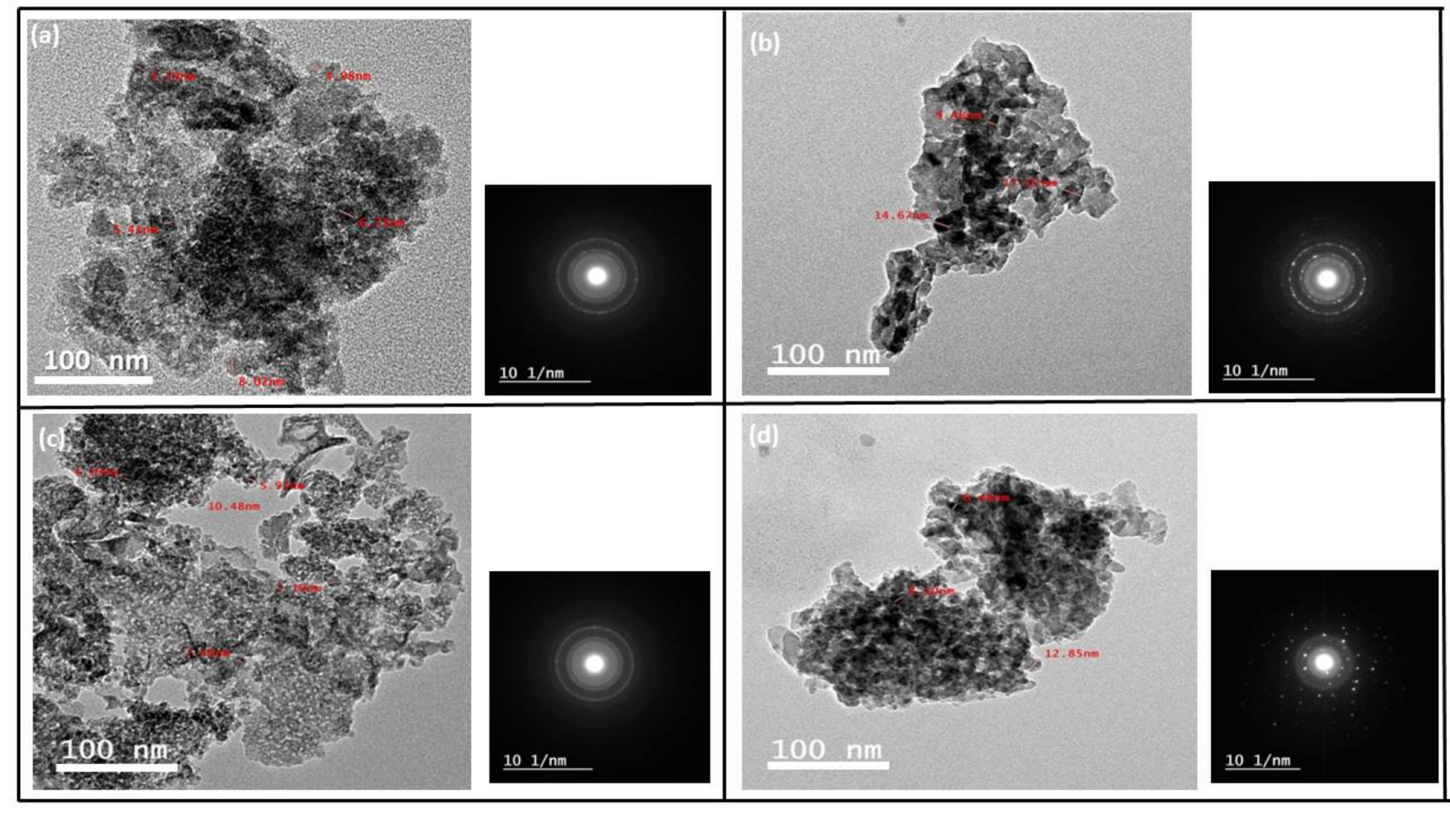
TEM images of Al_2_O_3_ powder prepared by polymeric method at temperature (**a**) 800 °C, (**b**) 1000 °C and alumina prepared by microwave method at temperature (**c**) 800 °C, (**d**) 1000 °C.

TEM images of alumina powder prepared by the polymeric method at different temperatures, 800 and 1000, are shown in Fig. 9a, b, respectively. The alumina prepared by microwave method is seen in Fig. 9c, d after firing at 800 and 1000 °C, respectively. It was observed that the prepared alumina powders by the microwave combustion method are more crystalline than those prepared by the polymeric method.

Alumina particles synthesized by microwave technique are presented in a nanosphere shaped at 800 °C and changed into nanoplate forms by raising the temperature to 1000 °C, while the alumina particles prepared via polymeric method at 800 °C still retained the shape of the amorphous polymeric structure that included some nanosphere grains compared to microwave method.

These findings are consistent with other studies that show a rise in calcination temperature is accompanied by a sequence of transformations, including—γ-Al_2_O_3_ → δ-Al_2_O_3_ → θ-Al_2_O_3_ → α-Al_2_O_3_^24^. All alumina phases that originated at low temperatures changed into α -Al_2_O_3_ at high temperatures. Low activation energies are required for transformations from one phase to another, whereas transformations proceed through nucleation, growth, and increased temperature^17,33,34^. The interaction of microwaves with the reactants at the molecular level, where this electromagnetic energy is transferred and transformed to heat by rapid kinetics through the motion of the molecules, can be used to explain why microwave heating causes the acceleration of the crystallinity and formation of alpha alumina at 1000 °C. Thus, the microwave combustion method causes the development of nanoparticles within a short period, the development of nanoparticles, early phase formation, and various morphologies^35–37^.

### Characterization of TiO_2_ substrate after coated with alumina coating solution

#### Phase composition of coated TiO_2_ substrate

**Figure 10:**
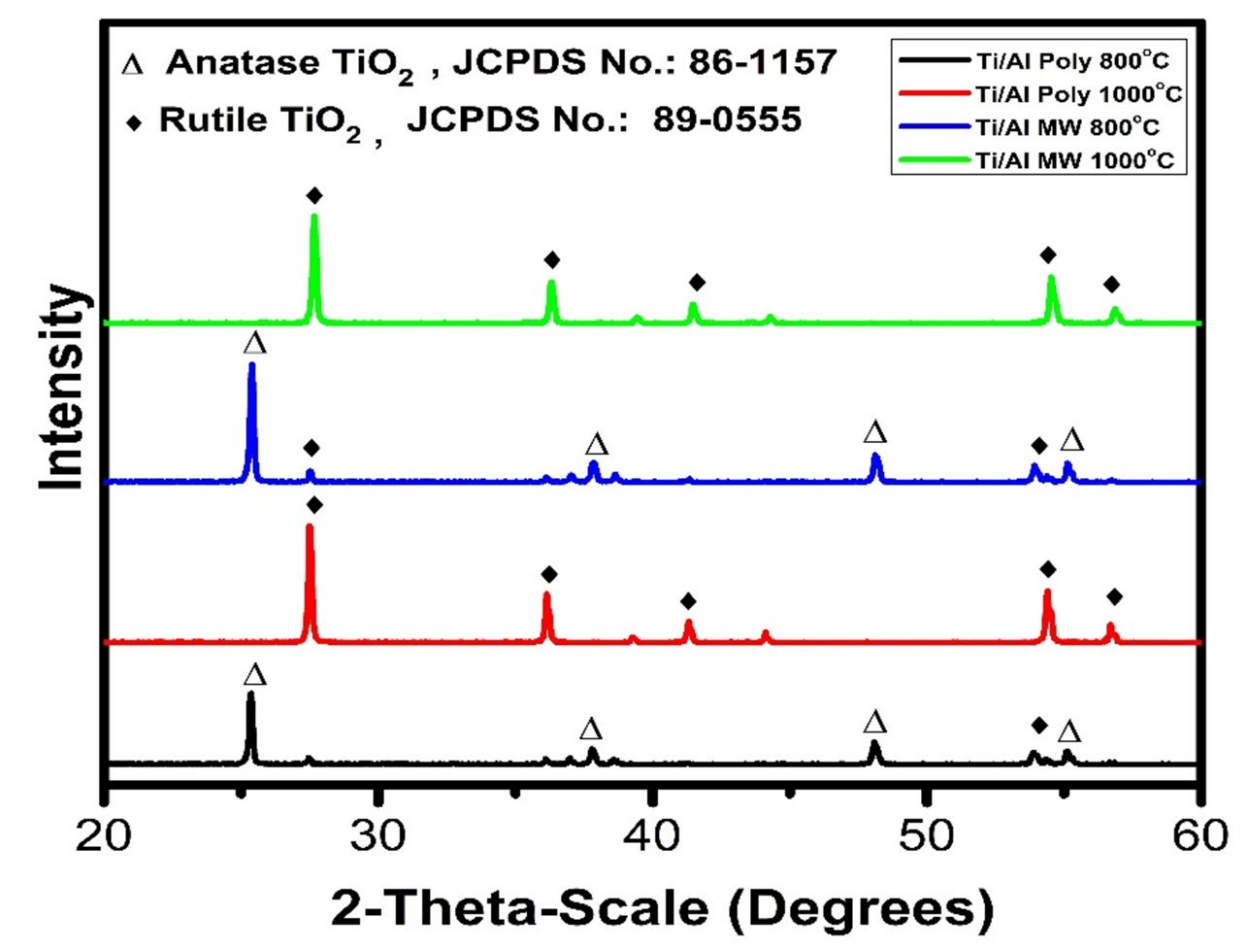
XRD patterns of TiO_2_ substrate coated with Al_2_O_3_ powder using polymeric (Poly) and microwave (MW) methods fired at 800 and 1000 °C.

The XRD patterns of the TiO_2_ substrate fired at 1000 °C and coated with Al_2_O_3_ by utilizing polymeric and microwave combustion methods, then firing at 800 and 1000 °C, are shown in Fig. 10. Through the XRD of TiO_2_-Al_2_O_3_ and its comparison with pure titanium substrate, which is seen in Fig. 3, an enhancement in the crystallization of TiO_2_ was observed by increasing the firing temperature. By firing the samples at 800 °C, the main anatase phase is indicated with small amounts of rutile phase. In comparison with pure substrate, the rutile peak intensities decrease. This can be attributed to the reaction between some γ-Al_2_O_3_ particles and rutile particles, leading to a small amount of aluminum titanate phase. However, the presence of the anatase phase at 800 °C as the main phase is due to some γ-Al_2_O_3_ particles surrounding the anatase phase. It prevents the growth of anatase crystals and their transformation to rutile at this temperature^38^. However, upon increasing the temperature to 1000 °C, the anatase phase disappears for the coated samples prepared by polymeric and microwave is observed. The rutile phase is the only phase obtained at this firing temperature compared with the pure substrate, as presented in Fig. 3. This can be attributed to the transformation of γ-Al_2_O_3_ to α-Al_2_0_3_ at 1000 °C, leading to the stability of the anatase phase. This work is conceded with Young Cheol Ryu^39^ observed that the metal cations diffusing from the substrate into the TiO_2_ layer might retard the Anatase–Rutile phase transformation of TiO_2_. The suppressing effect on the Anatase–Rutile transformation of TiO_2_ by mixed cations seems much stronger than that of single cations. In addition, it was observed that the Anatase –Rutile transformation in the TiO_2_ substrate deposited by α-alumina might proceed more easily, and the TiO_2_ substrate deposited by α-alumina has a higher rutile fraction^39^.

It was reported that the phase transformation of TiO_2_ depends on various parameters such as the initial particle size, impurity (doping) concentration, starting phase, and reaction atmosphere^39,40^. This work illustrates that the phase transformation of TiO_2_ from anatase to rutile is influenced by the metal ions (Al^3+^) diffused from the coated layer into TiO_2_ substrate as well as calcination temperature. Eskelinen et al.^41^, who studied the effects of heat treatment on the surface composition of TiO_2_ thin film in TiO_2_-phlogopite and in TiO_2_-muscovite system by X-ray photoelectron spectroscopy technique, claimed that the surface composition was dependent on the calcination temperature and the substrate components diffusing through the TiO_2_ film^39,41^.

#### IR analysis of coated TiO_2_ substrate

**Figure 11:**
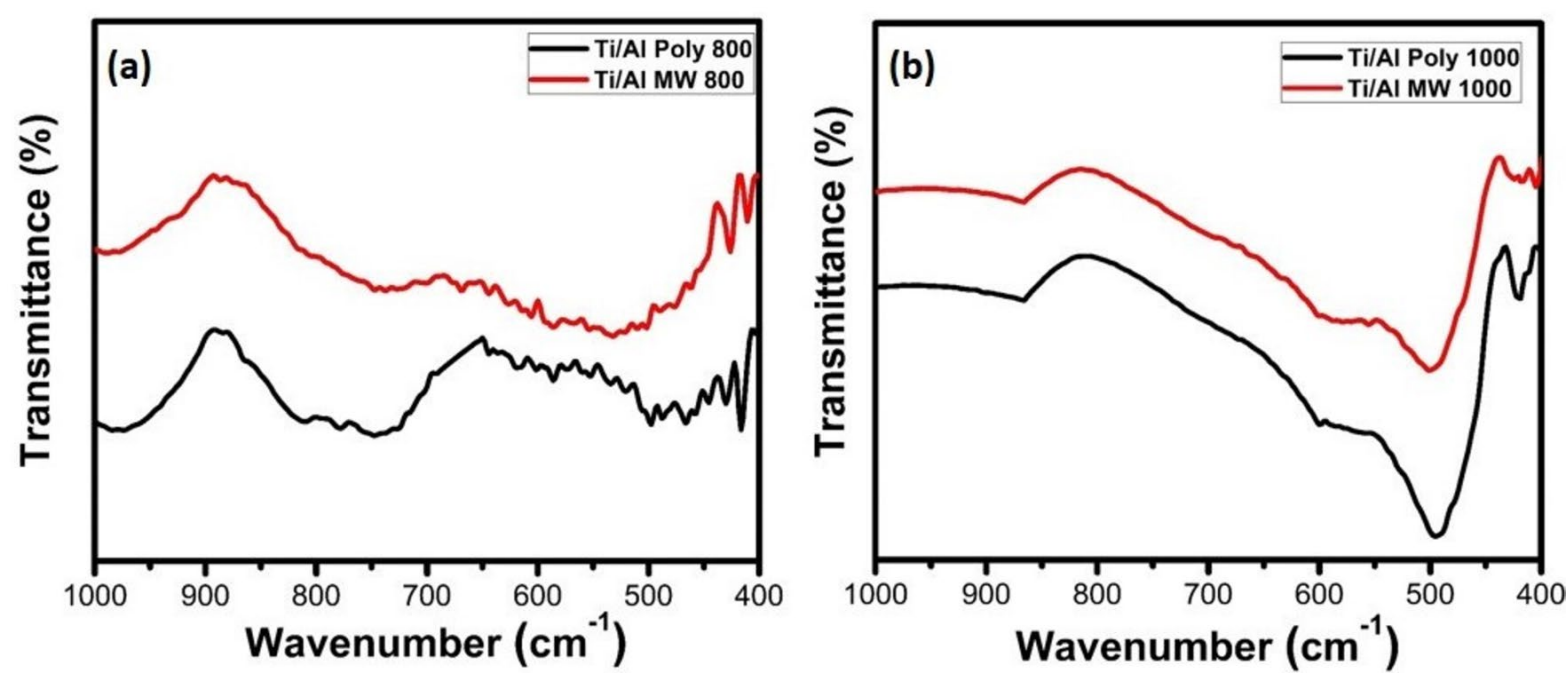
FTIR patterns of TiO_2_ substrate coated with Al_2_O_3_ powder by using polymeric (Poly) and microwave (MW) methods and fired at (**a**) 800 and (**b**) 1000 °C.

The IR patterns of the TiO_2_ substrate after being coated with Al_2_O_3_ using the polymeric method and microwave combustion method fired at 800 and 1000 °C are seen in Fig. 11a, b, respectively. In comparison, the IR analysis of the pure TiO_2_ substrate that fired at 1000 °C with TiO_2_ substrate coated with alumina, it was found that the band at 420 cm^−1^ is corresponding to anatase after firing at 800 °C as seen in Fig. 11a, while the bands at 499 and 850 cm^−1^ that showed in Fig. 11b after firing at 1000 °C refers to rutile phase. Bands related to Ti–O bond vibrations are found in the 493–579 cm^−1^ and 594–639 cm^−1^ ranges, as seen in Fig. 11a^42^. Bands caused by the stretching vibrations of the Al-O bonds of the octahedrally coordinated Al were seen in the 500–750 cm^−1^ range, whereas bands caused by the vibrations of the Al-O bond in AlO_4_ units are presented in the 750–900 cm^−1^ range, as seen in Fig. 11a^42^

#### Physical properties of coated TiO_2_ substrate

**Figure 12:**
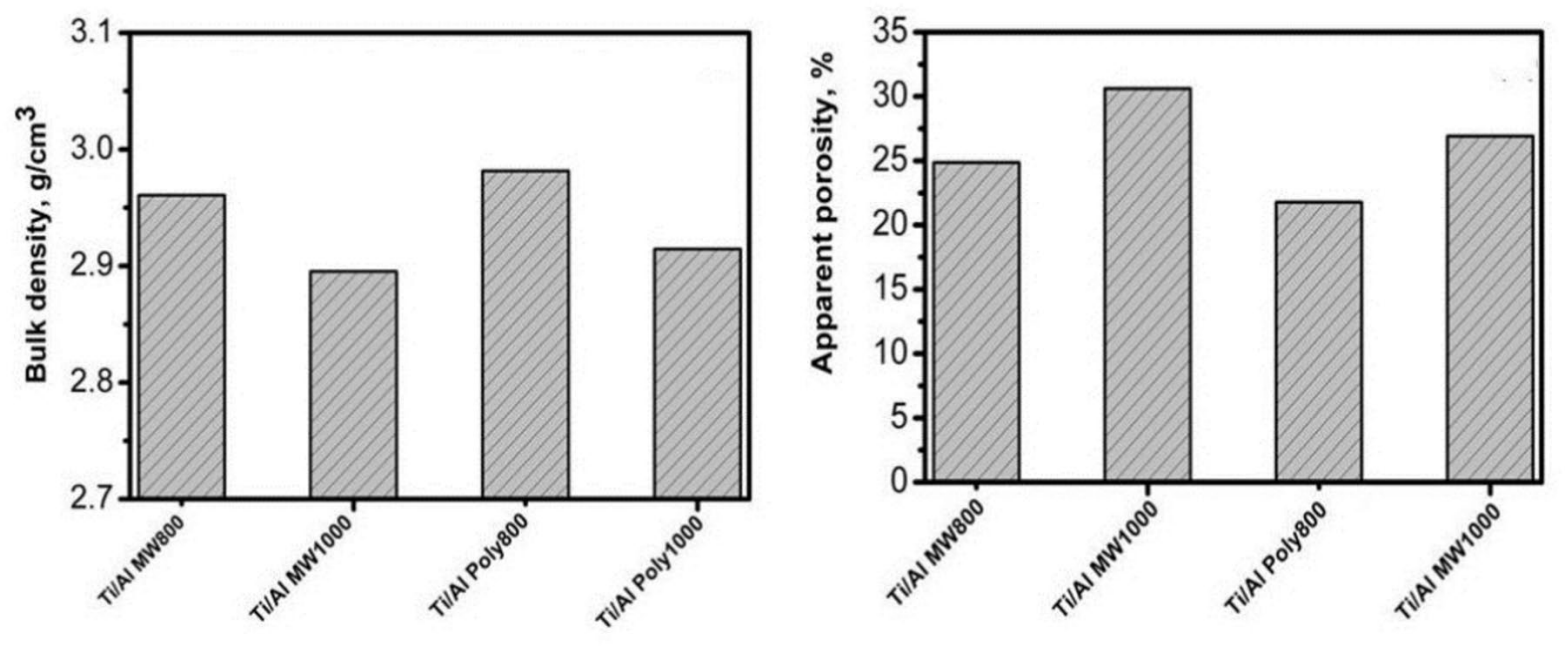
Physical properties of the coated TiO_2_ substrate with Al_2_O_3_ synthesized by polymeric (Poly) & microwave (MW) techniques and fired at 800 and 1000 °C.

Figure 12 depicts the physical characteristics of bulk density (BD) and apparent porosity (AP) of coated TiO_2_ substrate samples coated with alumina, created using polymeric and microwave techniques, and fired at 800 and 1000 °C temperatures. The apparent porosity increases and the bulk density decreases as the firing temperature rises, as was seen. Overall, all of the samples show pore characteristics. Porosity may result from several different factors, according to Yang^43^, including (1) gas formation during the sintering process and the subsequent expansion, entrapment, or escape of those gases, (2) shrinkage related to the sintering reaction (i.e., products with a higher specific volume than the starting materials), or (3) residual initial porosity of the powder due to partial sintering. When comparing titania-coated substrates with alumina made using the polymeric approach to those made using the microwave method, it was discovered that the microwave method produced coated substrates with greater porosity than the polymeric method. This is attributed to the absorption of Al_2_O_3_ into TiO_2_ samples using the polymeric method, which is higher than the microwave method and is indicated by the decrease in porosity. Another reason for this phenomenon is the higher crystallinity of the alumina produced by microwave as opposed to the polymeric method enhances the formation of some secondary aluminum titanate phase (Al_2_TiO_5_), which is difficult to sinter and has a lower density of 3.7 g/cm^3^ compared to that of titania at 4.23 g/cm^3^^44^. Due to the presence of an Al_2_TiO_5_ phase that is less to be detected in XRD (Fig. 10), high-temperature firing could not improve the densification percentage^44^. Additionally, the open spinel structure of γ-Alumina, a metastable phase known to include more different doping elements Ti^4+^ than α-alumina does^45^, which affects the grain boundaries (GB) diffusion properties, may be the cause of the decrease in porosity of the coated samples at 800 °C. *γ* → α Phase transition is described as a type of nucleation and growth transformation that affects the porosity quantities in the samples, according to S. Lartigue-Korinek et al.^45^.

#### SEM of the uncoated and coated TiO_2_ substrates with nano-Al_2_O_3_

**Figure 13:**
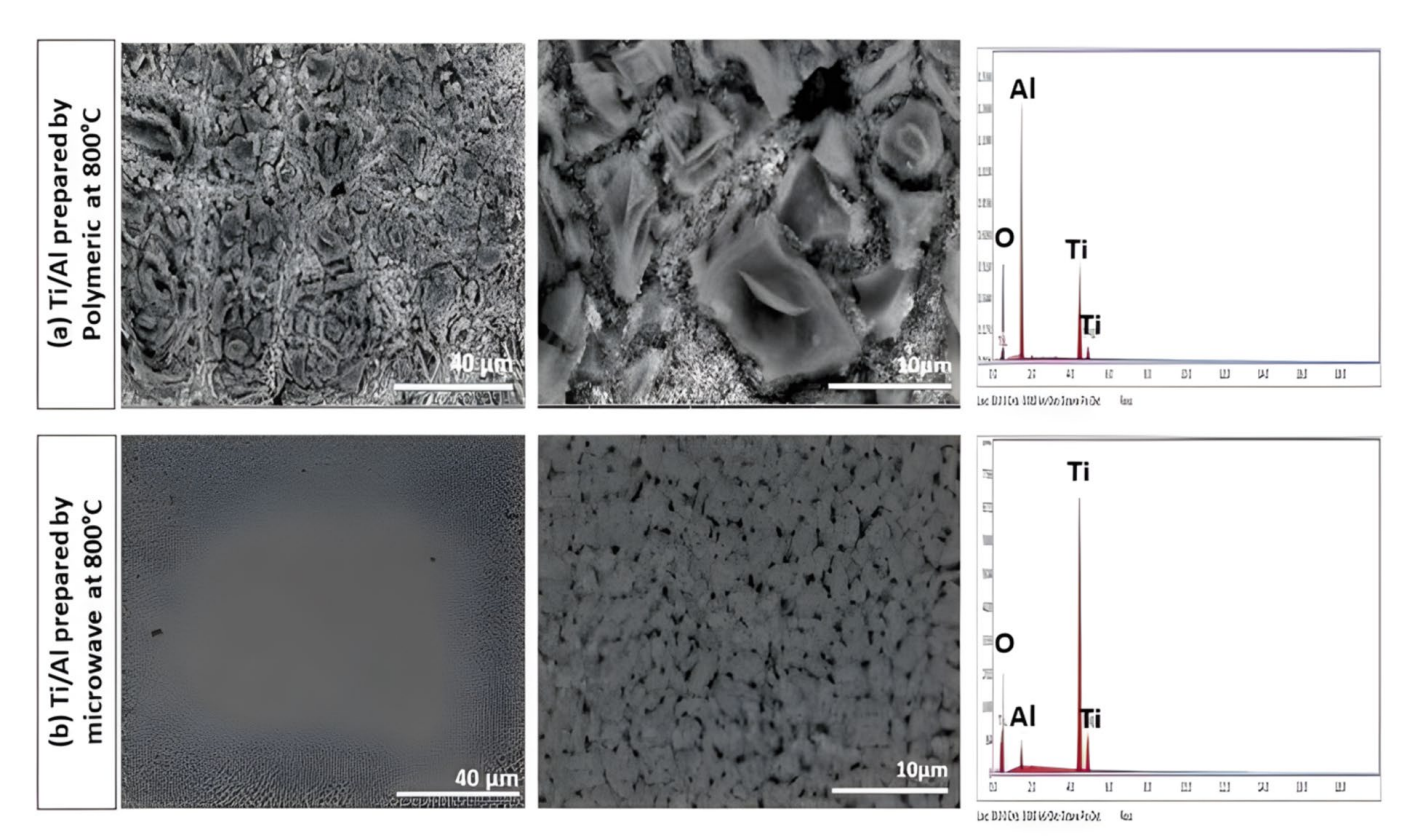
SEM of coated TiO_2_ substrates with Al_2_O_3_ synthesized via (**a**) polymeric and (**b**) microwave techniques and fired at 800 °C at different magnifications.

The SEM images of TiO_2_ coated substrate with Al_2_O_3_ synthesized via polymeric and microwave techniques and fired at 800 °C at different magnifications are shown in Fig. 13. It was observed that the surface of the coated TiO_2_ substrate by alumina prepared via microwave method has larger pinholes than the coated substrate with alumina prepared by polymeric method. In addition, the EDX analysis shows more absorption of Al ion for coated samples prepared by the polymeric method than the microwave method. This is attributed to the Al_2_O_3_ nanoparticles entering the TiO_2_ substrate's pores and filling them. At the same time, the rest of the nano-Al_2_O_3_ particles covered the TiO_2_ substrate surface well, increasing the Al ion absorption and decreasing the Ti ion absorption to a high extent. This is due to the crystallinity of prepared alumina by microwave (α-alumina) being higher than that prepared by polymeric method (γ-alumina), as shown previously in TEM result Fig. 9, as the metal ions with large atomic radii diffused less readily than those with smaller atomic radii^46^.

**Figure 14:**
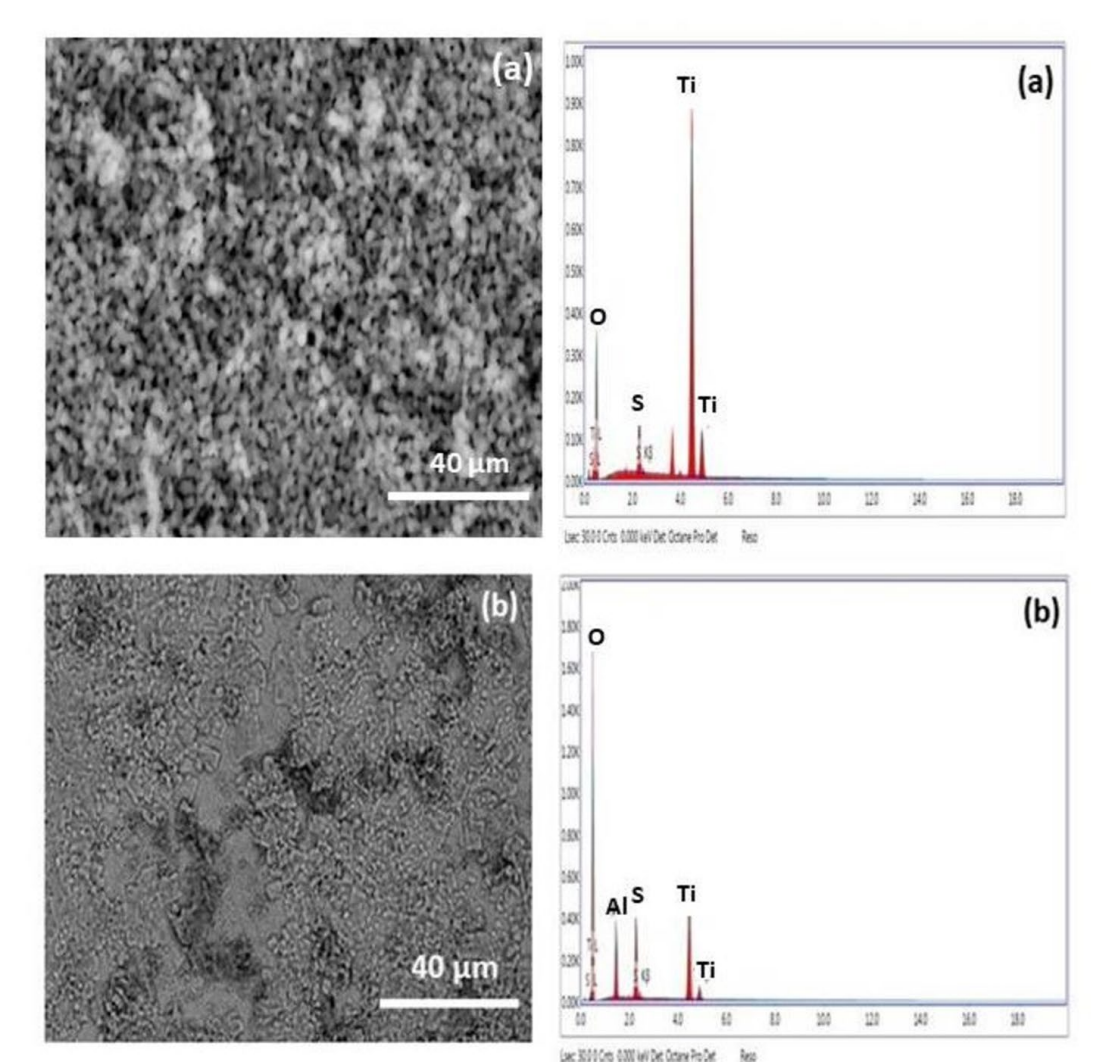
SEM image and EDX analysis after the PDP process of (**a**) the uncoated TiO_2_ substrate in 0.5 M H_2_SO_4_ solution and (**b**) the coated TiO_2_ substrate with nano-Al_2_O_3_ prepared by polymeric method in 0.5 M H_2_SO_4_ solution.

The SEM images of TiO_2_ substrates uncoated and coated with nano-Al_2_O_3_ synthesized via polymeric technique and fired at 800 °C after they were exposed to 0.5 M solution of H_2_SO_4_ are shown in Fig. 14a, b, respectively. Figure 14a illustrates the shape of the uncoated TiO_2_ substrate after the PDP was carried out in the aggressive medium 0.5 M H_2_SO_4_, It was clear that the surface of the sample contains many holes and roughness, and these characteristics are due to the effect of the solution.

EDX analysis and its data tabulated in Table 2 found that the weight percentage of titanium in the substrate before the PDP was executed was about (54%). In comparison, it became (44%) after the PDP was achieved. This is due to the presence of sulfur element from the (SO_4_^2−^) group, as its weight percentage is recorded at about 3%; thus, it penetrated the surface of the substrate, which led to a reduction in the weight percentage of titanium. Also, the effect of the penetration of sulfur ions to the surface of the substrate is demonstrated by increasing the porosity of the uncoated TiO_2_ substrate from 30% before PDP to 33% after PDP, as was established in Table 3, which means that the surface of the sample had been damaged and has many pores.

**Table 2:** Element composition of uncoated and coated TiO_2_ substrate by alumina prepared by (Poly) method at 800 °C before and after the PDP process in 0.5 M H_2_SO_4_ solution.

	Element	Weight %	Atomic %
Uncoated TiO_2_ substrate before PDP	O	45.9	71.76
	Ti	54.1	28.24
Uncoated TiO_2_ substrate after PDP	O	52.23	75.98
	Ti	44.40	21.58
	S	3.37	2.44
	Al	–	–
Coated TiO_2_ substrate before PDP	O	45.65	70.90
	Ti	52.08	27.02
	S	–	–
	Al	2.27	2.09
Coated TiO_2_ substrate after PDP	O	70.04	84.67
	Ti	17.25	6.96
	S	6.58	3.97
	Al	6.13	4.39

**Table 3:** Effect of the porosity percentage on the corrosion rate of the TiO_2_ substrate uncoated and coated by alumina by polymeric (Poly) and microwave (MW) methods after tested in 0.5 M H_2_SO_4_ solution.

Sample	Degree of Temp.	Porosity%	C.R., mm/year
Uncoated TiO_2_ before PDP	–	30.7692	–
Uncoated TiO_2_ after PDP	–	33.6541	67.71
Coated TiO_2_ (Poly) before PDP	800	21.1035	–
Coated TiO_2_ (MW) before PDP	800	24.8599	–
Coated TiO_2_ (Poly) after PDP	800	23.0012	16.30
Coated TiO_2_ (MW) after PDP	800	27.799	29.75

Figure 14b represents the coated TiO_2_ substrate with nano-Al_2_O_3_ prepared by the polymeric method and calcined at 800 °C after being exposed to the aggressive medium 0.5 M H_2_SO_4_ solution and tested by the PDP method. It can be seen that the surface of the substrate no longer has pinholes, and the percentage of the surface porosity was calculated; it was found to be about 23% (Table 3); this means that the presence of the nano-Al_2_O_3_ helped to coat the surface well, which stimulated the reduction of pores. In addition, it can be noticed that the surface of the sample became free from cracks and gaps without scratches and also smoother. This result proves the alumina's effect in protecting the sample's surface^47^. The presence of a few pores in the range of nano-scale on the sample's surface is a good factor in forming an oxygen barrier as a protective layer for the substrate in the corrosive medium^48^. The data obtained from EDX analysis showed that the percentage weight of the oxygen is about 70%, and this result explains the formation of an oxygen barrier, and the high percentage weight of oxygen may be related to the reduction of sulfate ions^49^.

#### Compressive strength for the uncoated and coated TiO_2_ substrate

**Figure 15:**
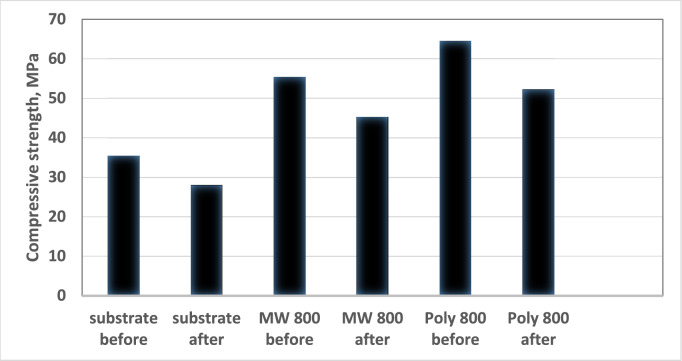
Compressive strength values for uncoated and coated titania substrates before and after exposure to sulfuric acid treatment.

Upon calcination at 800 °C, a few selected coated samples are examined for compressive strength and contrasted with uncoated substrate samples. Unlike microwave-coated samples and uncoated substrates, the TiO_2_-coated substrates with alumina via polymeric approach exhibit the highest strength among the remaining samples, with a maximum strength of about 65 MPa. This can be attributed to some of the Al_2_O_3_ nanoparticles entering the titania substrate's pores and filling them while the rest of the nano-Al_2_O_3_ particles well covered the titania substrate surface, as discussed previously in the SEM results; this behavior led to an increase in the strength of the TiO_2_ coated substrates compared to uncoated TiO_2_ substrates. The polymeric approach Al_2_O_3_ nanoparticles' tiny particle size with appropriate distribution produces the maximum reinforcing effect compared to the uncoated TiO_2_ substrate samples^50^. They are absorbed by the titania substrates, which reduces the titania substrate porosity compared to the alumina prepared via the microwave process, which has the opposite effect on strength values. Additionally, it has been found that treating the uncoated titania substrates with sulfuric acid decreased their compressive strength values to approximately 28 MPa, while treating the coated titania substrates with alumina prepared via microwave and polymeric method with sulfuric acid decreased their compressive strength value from 55 and 65 to be 45 and 52 MPa, respectively. As presented in Fig. 15.

#### Corrosion test estimation

**Figure 16:**
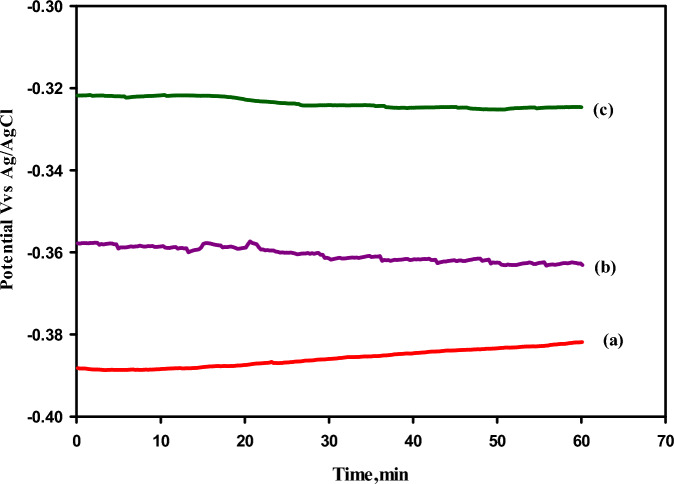
Open circuit potential of TiO_2_ substrate in 0.5 M H_2_SO_4_ solution (**a**) the substrate without coating (**b**) the substrate after coated by alumina prepared by (MW), method (**c**) the substrate after coated by alumina prepared by (poly), method examined in 0.5M solution of H_2_SO_4_ at 25 °C.

Open circuit potential measurements. The open circuit potential of the uncoated and coated TiO_2_ substrate with alumina (Al_2_O_3_), which was prepared by polymeric (poly) and microwave (MW) methods at 800 °C, was measured in the aggressive medium 0.5M solution of H_2_SO_4_. The experimental results of the OCP were presented in Fig. 16 as three curves that have analogous shapes with an approximate difference: (a) the substrate without coating, (b) the substrate coated with alumina by the (MW) method, and (c) the substrate coated with alumina by the (poly) method. It can be seen from Fig. 16 that the OCP was shifted in a positive direction, from about − 0.382 V for the uncoated TiO_2_ substrate to about − 0.363 V for the coated TiO_2_ substrate with alumina, which was prepared by (MW) method and − 0.321 V for the coated TiO_2_ substrate with alumina which prepared by (poly), method against silver/silver chloride. This result was attributed to forming a protective coating layer on the surface of the TiO_2_ substrate. As well as this result also confirms that the polymeric method for coating was better than the microwave method, and this result agrees with the above discussion.

Potentio-dynamic polarization method. The PDP experiment is one of the successful methods used to determine the corrosion rate of the substrate exposed to aggressive media like sulfuric acid, nitric acid, hydrochloric acid, and sodium chloride and the inhibition efficiency of the inhibitor or the coating which used to hinder the corrosion process^51^. The coating process is considered one of the important methods to increase the efficiency of some materials by changing some of their physical properties to protect the metals and their alloys from the undesirable effects of the corrosion process^52^. Among the materials used in this field are ceramic materials such as Al_2_O_3_, ZrO_2_, and TiO_2_^53^. According to the data and the results of the PDP experiments for the TiO_2_ substrates uncoated and coated with alumina (Al_2_O_3_), after exposing them to a 0.5 M solution of sulfuric acid as an aggressive medium, it was found that the corrosion rate of the uncoated substrate was decreased from the value (67.71 to 16.30 and 29.75 mm/year), after it was coated with Al_2_O_3_ by polymeric (poly), and microwave (MW), methods and calcined at 800 °C, respectively. By comparing the corrosion rate values of the two coating methods, it can be noticed that the polymeric method was better than the microwave method. This result may be attributed to the shape of the phase formed by alumina on the substrate after it was coated, as was reported in previous studies^54,55^. The analysis and characterization of the TiO_2_ substrate explained that, after the substrate was coated with alumina, which was prepared by the microwave method at a temperature of 800 °C, the coating layer was formed in the alpha form of aluminum oxide crystallized in the corundum structure, which has some properties such as low surface area and is almost non-porous^56^. The coating layer of alumina prepared by the polymeric method at the same temperature was formed in the gamma alumina phase, which has some excellent properties, such as a large surface area with some porosity, which resulted in decreasing the corrosion rate of the substrate-coated by polymeric method more than the substrate coated by microwave method at 800 °C, as shown in Table 4^57^.

**Table 4:** The parameters of the PDP process of uncoated and coated TiO_2_ substrates with Al_2_O_3_ prepared by microwave method (MW) and polymeric method (Poly) in 0.5 M solution of H_2_SO_4_ at 25 °C.

	T (°C)	β_a_, made^−1^	β_c_, mV dec^−1^	− E_corr_ mV	${i}_{corr}^{o}$ μA cm^−2^	*IE*%	C.R., mm/year
Substrate without coating	**–**	272.77	255.86	409.13	712.62	–	67.71
Substrate coating by (MW)	800	382.25	327.45	382.35	313.15	56.06	29.75
Substrate coating by (Poly)	800	193.39	376.07	289.73	152.79	78.56	16.30

Equation (1) was used to deduce the percentage of the inhibition efficiency of the substrate after it was coated with nano-Al_2_O_3_ to prevent the corrosion process, and all the parameters are established in Tables 4 and 5.

1 $$IE\%=\frac{{i}_{corr}^{o}-{i}_{corr}}{{i}_{corr}^{o}} \times 100$$

where ${i}_{corr}^{o}$ and ${i}_{corr}$ are the corrosion current density of the uncoated and coated substrate, respectively.

**Table 5:** The electrochemical impedance data of the TiO_2_ substrate uncoated and coated by nano-Al_2_O_3_ prepared by microwave method (MW) and by polymeric method (Poly) tested in 0.5 M H_2_SO_4_ at 25 °C.

	T (°C)	R_s_ Ω cm^2^	R_ct_ Ω cm^2^	CPE_1_		R_f_ Ω cm^2^	CPE_2_		Rp Ω cm^2^	*IE*%
				*Y*_o_ μΩ cm^−2^	*n*		*Y*_o_ μΩ cm^−2^	*N*		
Substrate without coating	–	15.089	53.800	667.012	0.443	12.981	450.521	0.442	66.781	–
Substrate coating by (MW)	800	70.412	120.815	215.043	0.525	25.340	115.000	0.447	146.155	54.308
Substrate coating by (Poly)	800	26.614	178.033	98.108	0.983	105.390	28.406	0.445	283.423	76.437

**Figure 17:**
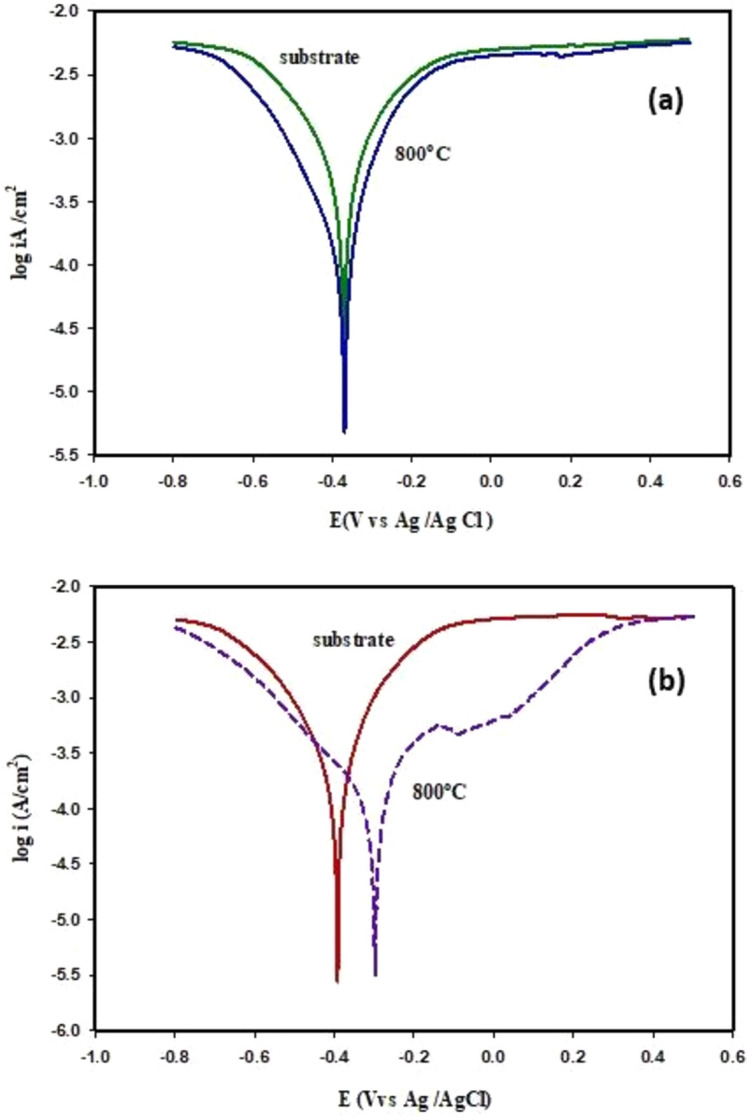
Tafel plots of uncoated and coated TiO_2_ substrate by the nano-Al_2_O_3_ after PDP process (**a**) coating by microwave method (MW) (**b**) coating by polymeric method (Poly), examined in 0.5 M solution of H_2_SO_4_ at 25 °C.

Good values of the inhibition efficiency (56% and 78%) of the working electrode (TiO_2_ disc), after it was coated with alumina, which was prepared at 800 °C by microwave and polymeric methods and tested in the corrosive medium (0.5 M H_2_SO_4_), can be interpreted by reducing the active area on the substrate due to the formation of mix film of Al_2_O_3_, TiO_2_ phase^58^ and TiAl phase; which it has excellent mechanical properties and corrosion resistance at temperatures (over 600 °C)^59^. The appearance of smooth polarization curves in Fig. 17a, b and only one peak at the corrosion potential is evidence that the formed film was electrochemically inactive^60^. This result agrees with the previous characterization of the surface. It was clear from the results reported in Table 4 that the value of the corrosion potential (*E*_corr_), of the uncoated substrate (TiO_2_ disc), recorded − 409.13 mV, shifts to more noble value in a positive direction after coating with Al_2_O_3_ to reach − 289.73 mV at (800 °C), for the sample coated with alumina by polymeric method, while the corrosion potential (*E*_corr_), recorded − 382 mV, when the sample coated with Al_2_O_3_ by microwave method at (800 °C); this result verifies that the use of the polymeric method (Poly), for coating was better than the microwave method (MW). Also, this result confirms the formation of a good coating layer of the nano-composite formed of alumina and titanium (Al_2_O_3_ TiO_2_ phase and TiAl phase) that exhibits good resistance against the corrosion process^61^. As mentioned by M. Sabzi et al., the potentiodynamic polarization diagram for galvanized steel in seawater environment electrolyte shows that the galvanized layer's resistance to corrosion is greater than that of the steel underlying. Moreover, the impedance resistance of galvanized steel decreased as the surrounding temperature rose^62^.

Through Table 4, it can also be noted that the corrosion current density (${i}_{corr}^{o}$), was decreased from the value of 712.62 μA cm^−2^ of the uncoated TiO_2_ substrate to reach a value of about 313.15 and 152.79 μA cm^−2^ of the coated substrate by the nano alumina that, synthesized by microwave and polymeric methods, respectively. This result indicates that the formed film increases the ability of the substrate to resist the corrosion process^2^.

**Figure 18:**
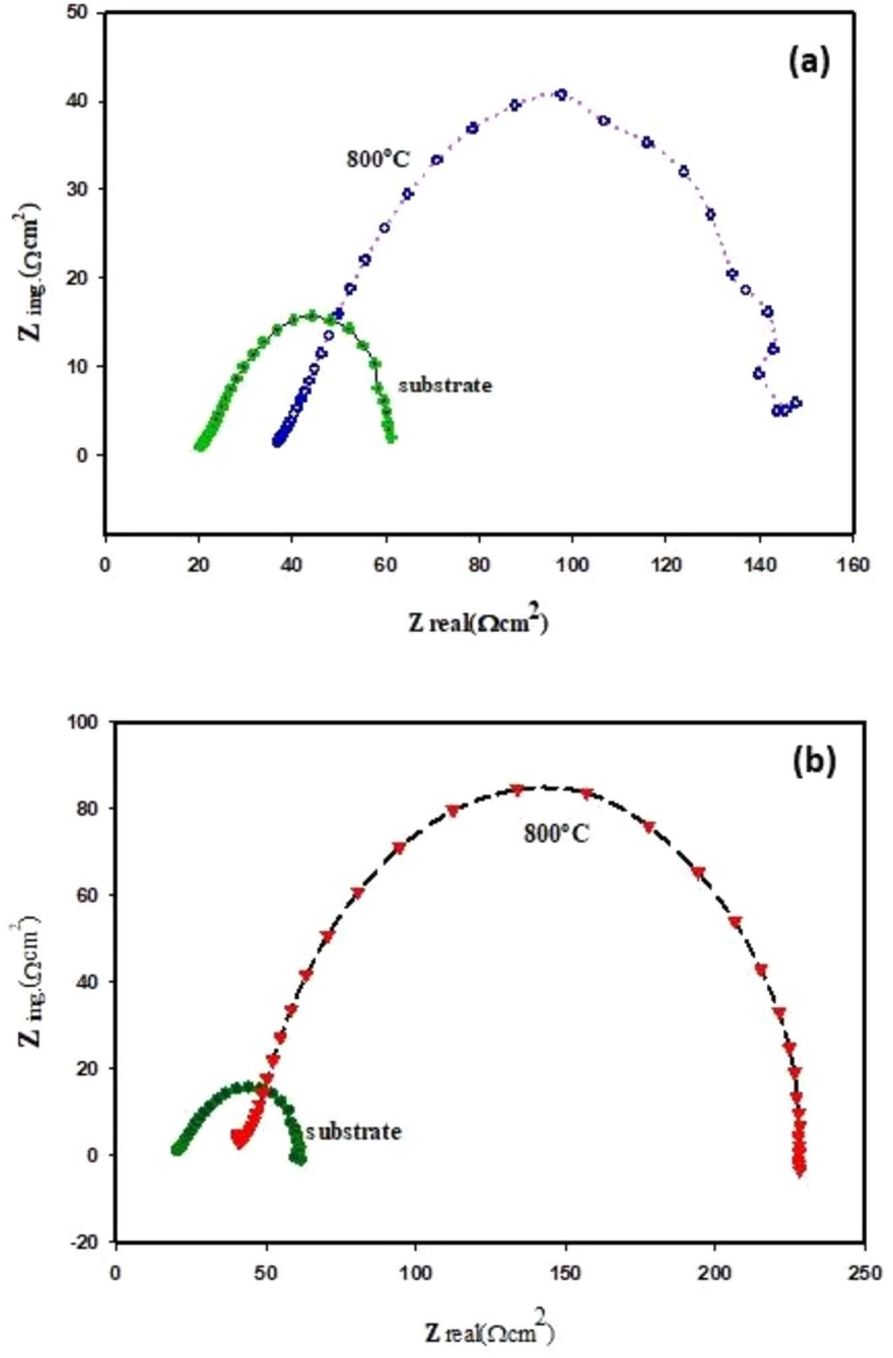
Nyquist plots (**a**) TiO_2_ substrates uncoated and coated with nano-Al_2_O_3_ prepared by microwave method (MW), (**b**) TiO_2_ substrate uncoated and coated with nano-Al_2_O_3_ prepared by polymeric method (Poly) tested in 0.5 M H_2_SO_4_ at 25 °C.

**Figure 19:**
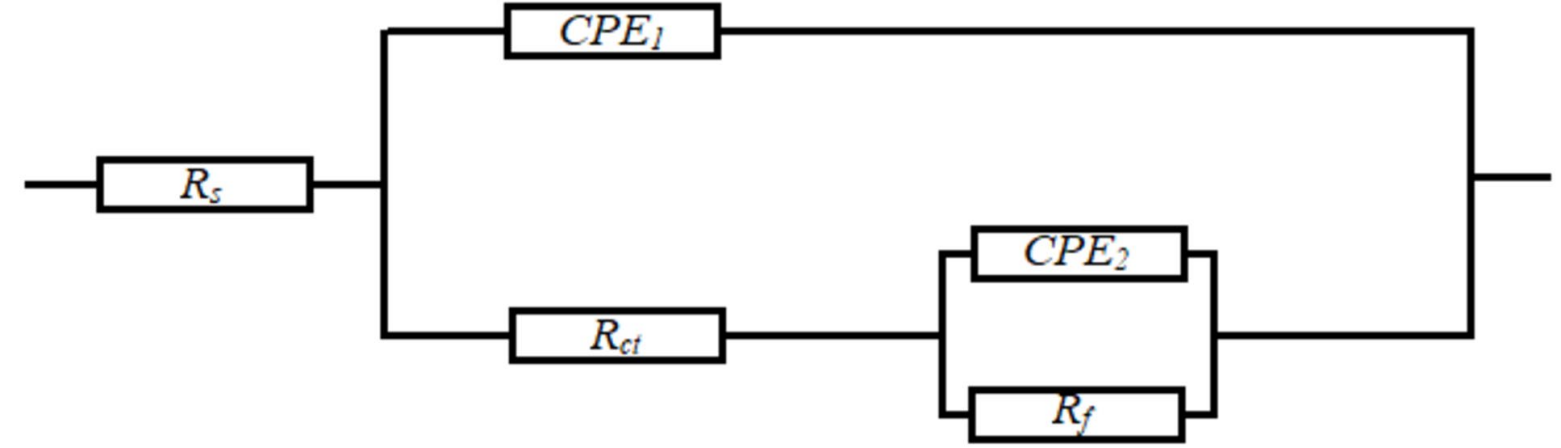
The modal of the equivalent circuit used to fit the impedance data.

**Figure 20:**
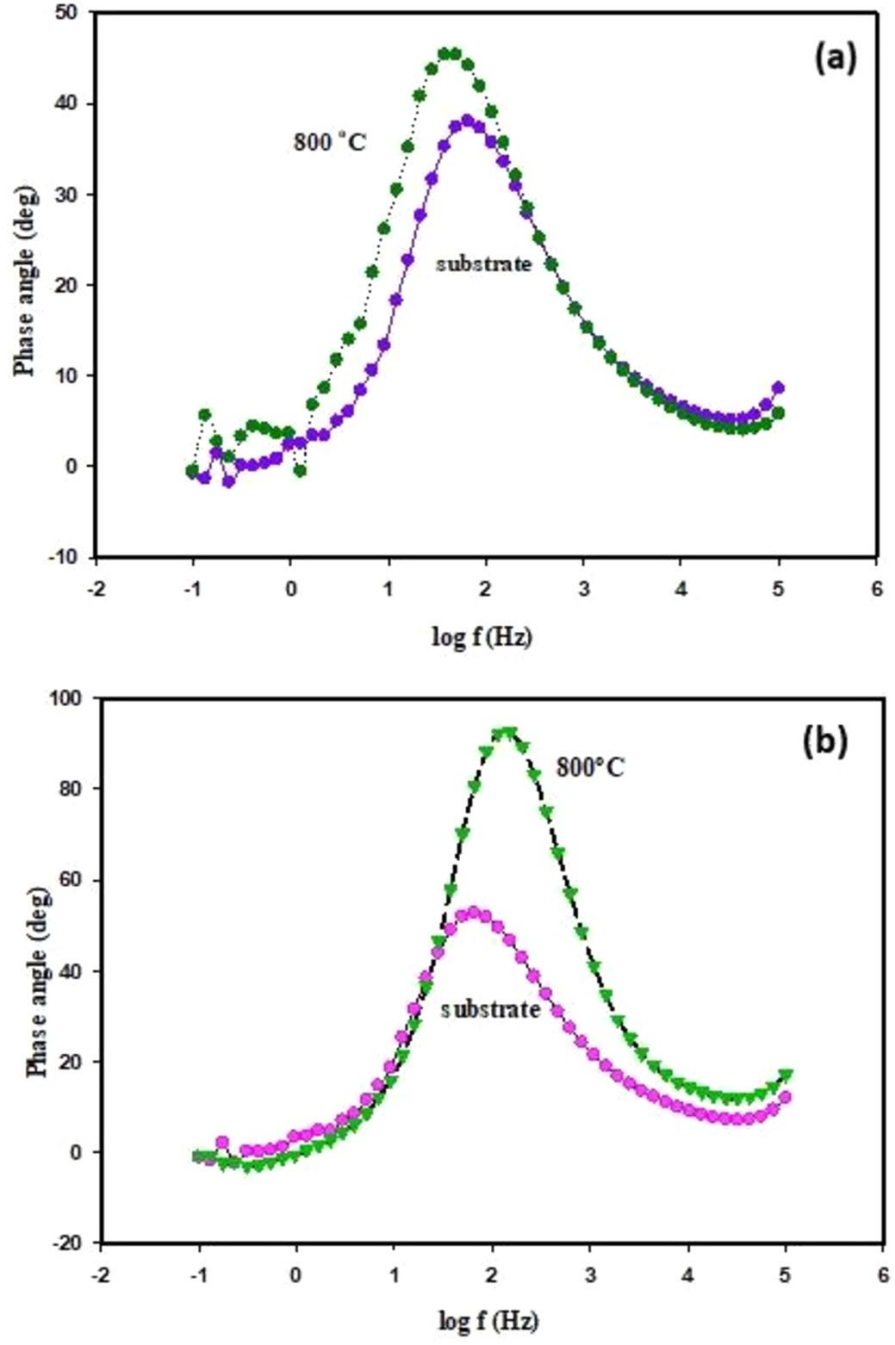
Bode phase of the impedance data (**a**) TiO_2_ substrates uncoated and coated with (Al_2_O_3_), formed by microwave method (MW), (**b**) TiO_2_ substrates uncoated and coated with (Al_2_O_3_), formed by polymeric method (Poly), tested in 0.5 M solution of H_2_SO_4_ at 25 °C.

**Figure 21:**
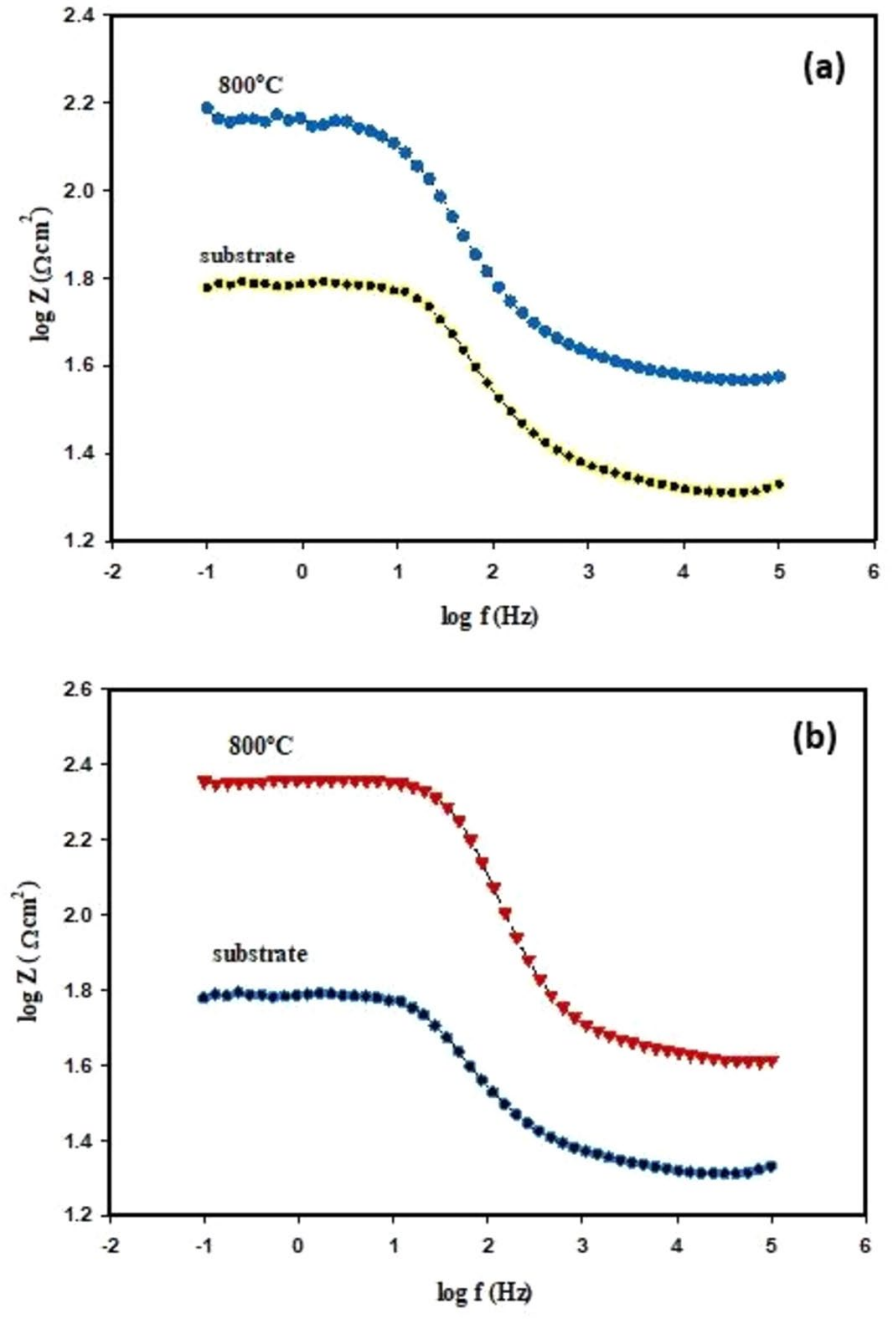
Bode modulus of the impedance data (**a**) TiO_2_ substrates uncoated and coated with (Al_2_O_3_), formed by microwave method (MW), (**b**) TiO_2_ substrates uncoated and coated with (Al_2_O_3_), formed by polymeric method (Poly) tested in 0.5 M solution of H_2_SO_4_ at 25 °C.

Electrochemical impedance spectroscopy method. The EIS diagrams of the uncoated and coated substrate (TiO_2_ disc), after tested in 0.5 M H_2_SO_4_ solution, are depicted in Figs. 18, 19, 20 and 21. It can be seen from Fig. 18a, b that the Nyquist plots deviated from the ideal semicircle, meaning that it appeared as a depressed semicircle. This phenomenon, the effeteness of the frequency dispersion, has been attributed to forming a passive film with microporous, roughness and irregularity of the surface of the substrate, and random distribution of the active sites^63,64^.

Increasing the diameter size of the semicircle of the substrate (TiO_2_ disc) after it was coated with alumina (Al_2_O_3_) pointed to the formation of a good passive layer on the surface of the sample attributed to the presence of alumina, which exhibited good stability in various media; as it allows improving the corrosion resistance of the sample surface^65^. In addition, the comparison between the diameter size of the two capacitive loops of the substrate (TiO_2_ disc), after it was coated with alumina, which was prepared by microwave and polymeric methods Fig. 18a, b, respectively, showed that; the diameter size of the capacitive loop Fig. 18b, was larger more than that of Fig. 18a, this result agrees with the above discussion that the polymeric method, was better for coating than the microwave method. According to previous studies, the large diameter of the semicircle of the sample also means more protection against the effects of the corrosion process^66^.

The percentage of the inhibition efficiency (*IE*%) of the coating material (Al_2_O_3_) was calculated using. ${R}_{ct}$ according to the following equation:

2 $$IE\%=\frac{{{R}_{ct}^{o}-R}_{ct}}{{R}_{ct}^{o}} \times 100$$

Abbreviation ${R}_{ct}^{o}$ and ${R}_{ct}$ refers to the charge transfer resistance of coated and uncoated samples after the EIS test. It was cleared by reading the results reported in Table 5 that the charge transfer resistance. ${R}_{ct}$ increases after the substrate (TiO_2_ disc) is coated with nano-(Al_2_O_3_); this result indicates that the corrosion rate is reduced due to the formation of a protective film of the coating materials on the surface of the sample.

The equivalent circuit used for the analysis of EIS data was [R(QR)(QR)], shown in Fig. 19, where (*R*_*s*_) is the solution resistance, (*R*_*ct*_) the charge transfers resistance and (*R*_*f*_) the film resistance. Due to the presence of the phenomenon frequency dispersion; the constant phase element (*CPE*), was used in the equivalent circuit instead of the double-layer capacitance (C_*dl*_), to be more suitable for the results of the impedance data^67^.

The constant phase element (*CPE*), was determined from the following equation:

3 $${Q}_{CPE}={\text{Y}}_{\rm o}^{-1}({\text{j}} \upomega)^{-{\text{n}}}$$

According to the above equation, the constant phase element (*CPE*) consists of a constant Y_o_, j is an imaginary number equal (− 1)^1/2^, ω is the angular frequency in rad/s, and a component *n* is the exponent of *CPE* expresses phase shift where *n* is between (− 1) and (+ 1), i.e. (− 1 ≤ *n* ≤  + 1). So, if a component *n* = 0, the constant phase element acts as a resistor, while *n* =  − 1, the *CPE* appears as an inductor, and if *n* =  + 1, the constant phase element performs as a capacitor^68^. Shifting of the phase angle or the component *n* to more positive value after the substrate (TiO_2_ disc), coated with nano-Al_2_O_3_, by microwave method from (0.443 to 0.525 at 800 °C) and after the substrate coated by polymeric method from (0.443 to 0.983 at 800 °C), this result refers to that, the irregularity of the sample surface was decreased^69^, as be reported in Table 5, and can also be seen in Fig. 20a, b.

Also, it can be observed from Table 5 that the value of *n* is less than 0.5 for the substrate without coating; this result means that the corrosion rate was controlled by the diffusion process, while after the substrate was coated, the value of *n* increased than 0.5 indicates that the corrosion rate controlled by the charge transfer resistance ${(R}_{ct}),$^70^. Figure 21a, b shows the Bode modulus impedance Z of the TiO_2_ substrate without and with coating by nano-Al_2_O_3_. It was cleared by looking at Fig. 21a, b, that; the Bode modulus increased from (1.8 to 2.4 Ω cm^2^) after the sample was coated, and this is explained by the good effect of the coating material (nano-Al_2_O_3_), to reduce the corrosion rate by slowing down the charge transfer resistance ${(R}_{ct}),$^71^. It can be noticed that the two electrochemical techniques, PDP and EIS, used in this study agree with each other.

## Conclusion

This work successfully created a nano-Al_2_O_3_ coated layer on TiO_2_ substrates using polymeric and microwave techniques to extend the ceramic field applications. The following results were observed:

1. A temperature of 1000 °C was found to be ideal for preparing the TiO_2_ substrate samples.
2. Phase composition of TiO_2_ substrate samples revealed that anatase and rutile phases were formed at 1000 °C.
3. The nano-Al_2_O_3_ coating layer was created using polymeric and microwave techniques. According to the findings, after firing at 800°C, the polymeric method produced more nano γ-Al_2_O_3_ than the microwave method.
4. Raising the temperature to 1000 °C increases the formation of α-Al_2_O_3_ production using the microwave method.
5. The formation of anatase at 800 °C and rutile at 1000 °C are caused by the Al_2_O_3_ deposition on the TiO_2_ substrate
6. At 800 °C as opposed to 1000 °C, the coated TiO_2_ substrates with Al_2_O_3_ showed superior mechanical and physical properties.
7. Good compaction was obtained using the polymeric method rather than the microwave method for preparing the Al_2_O_3_ coated layer on TiO_2_ substrates at 800 °C.
8. Following treatment with an aggressive sulfuric acid medium, coated TiO_2_ substrates with nano-Al_2_O_3_ had higher compressive strength than uncoated TiO_2_ substrates.
9. According to the results of the corrosion tests, the nano alumina (Al_2_O_3_) can be used as a good coating material to protect the TiO_2_ substrate against the effect of the corrosive medium 0.5 M solution of H_2_SO_4_.

## Data Availability

The datasets generated and/or analyzed during the current study are not publicly available because they are private, but are available from the corresponding author on reasonable request.
